# Enhancing Bioplastic Degradation in Anaerobic Digestion: A Review of Pretreatment and Co-Digestion Strategies

**DOI:** 10.3390/polym17131756

**Published:** 2025-06-25

**Authors:** Mohamed Shafana Farveen, Raúl Muñoz, Rajnish Narayanan, Octavio García-Depraect

**Affiliations:** 1 Department of Genetic Engineering, College of Engineering & Technology (CET), SRM Institute of Science and Technology, Kattankulathur, Chennai 603203, Tamil Nadu, India; sm3871@srmist.edu.in (M.S.F.); rajnishn@srmist.edu.in (R.N.); 2 Institute of Sustainable Processes, Dr. Mergelina s/n, 47011 Valladolid, Spain; raul.munoz.torre@uva.es; 3 Department of Chemical Engineering and Environmental Technology, School of Industrial Engineering, University of Valladolid, Dr. Mergelina, s/n, 47011 Valladolid, Spain

**Keywords:** anaerobic digestion, pretreatment, co-digestion, bioplastic, methane, biodegradation

## Abstract

The increasing production of bioplastics worldwide requires sustainable end-of-life solutions to minimize the environmental burden. Anaerobic digestion (AD) has been recognized as a potential technology for valorizing waste and producing renewable energy. However, the inherent resistance of certain bioplastics to degradation under anaerobic conditions requires specific strategies for improvement. Thus, in this review, the anaerobic biodegradability of commonly used bioplastics such as polylactic acid (PLA), polyhydroxybutyrate (PHB), polybutylene adipate-co-terephthalate (PBAT), polybutylene succinate (PBS), polycaprolactone (PCL), and starch- and cellulose-based bioplastics are critically evaluated for various operational parameters, including the temperature, particle size, inoculum-to-substrate ratio (ISR) and polymer type. Special attention is given to process optimization strategies, including pretreatment techniques (mechanical, thermal, hydrothermal, chemical and enzymatic) and co-digestion with nutrient-rich organic substrates, such as food waste and sewage sludge. The combinations of these strategies used for improving hydrolysis kinetics, increasing the methane yield and stabilizing reactor performance are described. In addition, new technologies, such as hydrothermal pretreatment and microbial electrolysis cell-assisted AD, are also considered as prospective strategies for reducing the recalcitrant nature of some bioplastics. While various strategies have enhanced anaerobic degradability, a consistent performance across bioplastic types and operational settings remains a challenge. By integrating key recent findings and limitations alongside pretreatment and co-digestion strategies, this review offers new insights to facilitate the circular use of bioplastics in solid waste management systems.

## 1. Introduction

Plastic pollution has emerged as a pressing global environmental challenge. Global plastic consumption is projected to rise sharply from 464 million tons in 2020 to 884 million tons by 2050, leading to an estimated 4725 million tons of plastics accumulating in the environment [1,2,3]. The COVID-19 pandemic further exacerbated the situation due to the high demand for disposable personal protective equipment [4]. Despite ongoing recycling efforts, plastic production continues to outpace the recycling rates, with projections indicating that global plastic waste could triple by 2060 under a business-as-usual scenario [5,6].

**Figure 1:**
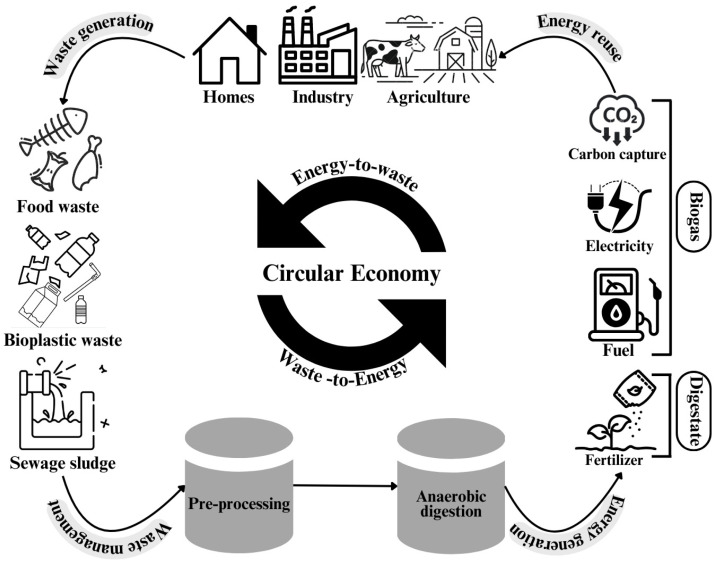
Integration of bioplastics and organic waste management within a circular economy framework through AD. Pre-processing improves degradation rates and energy recovery, contributing to carbon reuse and sustainable waste valorization.

As a response to the challenges posed by plastic pollution, bioplastics have garnered attention as a sustainable alternative in the context of a circular economy. The transition to bioplastics establishes a closed-loop system consistent with sustainability principles and resource efficiency and promotes an environmentally friendly approach to plastic production and disposal [7,8]. This concept is illustrated in Figure 1, which presents a comprehensive framework for integrating bioplastic waste streams into a circular economy via AD. This demonstrates the systematic transformation of diverse organic waste inputs to multiple value-added outputs, including biogas conversion to electricity and fuel and nutrient-rich digestate to fertilizer, thereby establishing complete resource circulation.

**Figure 2:**
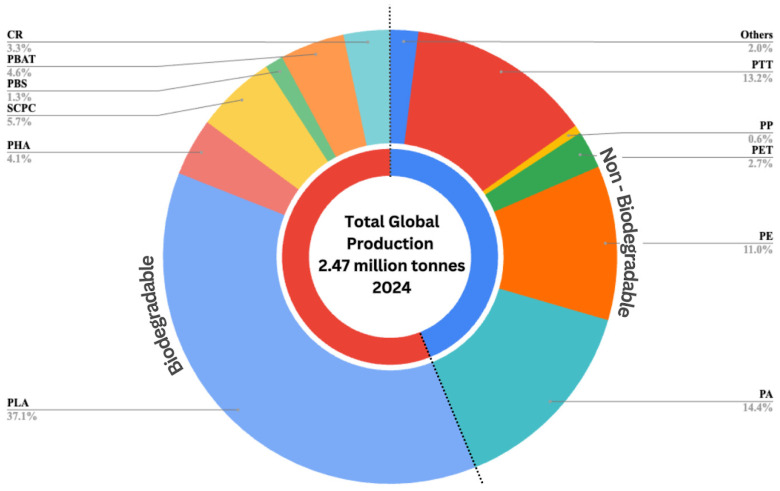
Global productions of bioplastics in the year 2024. Data released by European Bioplastics, Nova Institute 2024. PE: Polyethylene; PP: Polypropylene; PA: Polyamides; PET: Polyethylene terephthalate; PTT: Polytrimethylene terephthalate; PHA: Poly hydroxy-alkanoates; SCPC: Starch containing polycaprolactone; CR: Cellulose regenerated; PBS: Polybutylene succinate; PLA: Polylactic acid; PBAT: Poly(butylene adipate-co-terephthalate).

This circular economy vision is becoming increasingly viable as bioplastic production scales up globally. Bioplastic production has experienced remarkable growth, expanding 16 times faster than conventional plastics [9]. This growth is largely driven by key manufacturing regions, namely China, South Korea, the United States, the European Union, and Brazil, which collectively serve as the dominant global hubs for bioplastic production [10]. The scale of this expansion is demonstrated in Figure 2, which presents the breakdown of global plastic production in 2024, highlighting the growing contribution of bioplastics compared to conventional plastic types.

While bioplastics offer environmental advantages over conventional plastics, their rapid adoption creates new waste management challenges that require sustainable end-of-life (EoL) solutions. Thinking about possible effective means of waste management, recycling becomes critical in the circular economy of plastics, whereby the best recycling routes include alcoholysis, biological recycling, glycolysis, and pyrolysis [11]. Nevertheless, regarding the EoL management of biodegradable bioplastics, composting and AD are mature technologies that help control the bioplastic waste that cannot be properly recycled [12]. Among the waste-treatment methods, AD is attractive as a strategy for bioplastic valorization that allows for waste stabilization and renewable energy production. AD has more control over degradation, lower odor emissions, and has the advantages of biogas and nutrient-rich digestate extraction, in comparison to aerobic composting [13,14,15].

AD has been evaluated to some extent as an EoL method for biodegradable plastics, but the ubiquity of the microbial recalcitrance of many bioplastics to microbial hydrolysis produces suboptimal methane production and/or incomplete degradation. In order to overcome these limitations, recent studies have focused on process optimization strategies to increase the digestibility and energy recovery of bioplastics. Pretreatment methods, such as physical size reduction [16], thermal and hydrothermal treatments [17,18], alkaline treatment [19], and enzymatic pretreatment [20], have proved to be very promising for improving biopolymer solubilization, crystallinity removal, and hydrolysis rates. In the meantime, in co-digestion systems, the use of nutrient-rich substrates (such as food waste and sewage sludge) has shown synergistic effects, including enhanced microbial diversity, improved C/N ratio balance, increased process stability, and higher methane yields [21,22,23].

In recent years, there has been a notable increase in scientific interest surrounding the biodegradation of bioplastics across both managed environments, such as composting, AD and unmanaged settings like soil and aquatic systems. However, despite this expanding body of literature, comprehensive evaluations focusing on the practical challenges and operational limitations of bioplastics under AD remain relatively scarce. Addressing this gap, the authors present a critical and application-oriented analysis of the key factors influencing bioplastics degradation in AD systems. Moreover, the present review explores emerging strategies such as tailored pretreatments, microbial adaptation, co-digestion with readily degradable substrates, and process optimization that hold promise for enhancing the effectiveness and reliability of bioplastics management under anaerobic conditions. With the aim of consolidating existing knowledge on pretreatment and co-digestion methods and identifying optimal approaches to utilizing the full potential of AD as a sustainable solution for bioplastic waste management, this review provides a comprehensive analysis of two key aspects: (i) bioplastics compatibility for AD, where the biodegradability of different polymers under varying operational conditions is discussed, and (ii) process enhancement strategies, where recent research on pretreatment techniques and co-digestion methods are reviewed. The comprehensive review provided in this article can be used to tailor pretreatment and co-digestion methods to the specific needs of different bioplastic compositions to ensure a more sustainable and efficient AD process for bioplastic waste management. Additionally, an integrated approach that combines the enhancement strategies could be adopted to harness the full benefits and contribute to the development of circular economy principles in the bioplastics industry.

## 2. How Compatible Are Bioplastics with AD?

AD of bioplastics presents significant challenges, primarily due to the structural diversity and heterogeneous biodegradability of various polymer types. Efficient AD is not only based on the inherent biodegradability of the bioplastic but also on a series of operational parameters such as the temperature, particle size, inoculum-to-substrate ratio (ISR), and the chemical structure of the polymer [15,24]. While some bioplastics readily undergo hydrolysis and methanogenesis, others are resistant, resulting in a low methane yield and incomplete degradation. For example, Zhang et al. (2018) reported that just four cellulose-based materials among nine bioplastics tested by the authors showed remarkable biodegradability under anaerobic conditions, indicating significant differences in degradation capabilities among various plastics [25]. In addition, another study showed that the degradation of biodegradable bioplastics under thermophilic AD conditions may slow down when transferred to mesophilic conditions, suggesting challenges in achieving complete degradation [26]. These results underscore the necessity of a case-by-case assessment of the behaviour of bioplastics in AD. Therefore, the anaerobic digestibility of the most important commercial bioplastics, such as polylactic acid (PLA), polyhydroxybutyrate (PHB), poly(butylene adipate-co-terephthalate) (PBAT), polybutylene succinate (PBS), polycaprolactone (PCL), as well as starch-based bioplastics and cellulose-based materials, is critically reviewed in the following sections.

### 2.1. Polylactic Acid (PLA)

PLA is one of the most widely produced bioplastics due to its bio-based origin and favorable properties under industrial composting conditions. However, its anaerobic degradation behaviour is highly dependent on operational parameters. Under mesophilic conditions (~35–38 °C), PLA shows limited biodegradability, with substantial methane production requiring retention times exceeding 400 days to achieve the ultimate methane yield and reach around 74.7% degradation. In contrast, thermophilic digestion (~55 °C) markedly enhances PLA degradation, with significant biogas generation observed within 100 days [27,28]. Another study reported that PLA exhibited a methane yield of only 59 NmL CH_4_/g VS under mesophilic industrial AD, corresponding to minimal biodegradation (~11%). Using a multi-step modified Gompertz model, the authors predicted that the complete transformation of PLA into methane would require at least 200 days [29]. These findings are consistent with earlier reports that demonstrated enhanced PLA degradation under thermophilic conditions compared to mesophilic operation [30,31,32], reinforcing the critical role of temperature in accelerating biopolymer hydrolysis.

This temperature sensitivity is primarily attributed to PLA’s glass transition temperature (Tg) (~55–60 °C), beyond which the polymer chains become more mobile, allowing easier access for microorganisms and enzymes to break down the polymer [33]. Meanwhile, under mesophilic conditions, the polymer chains remain rigid and less susceptible to degradation [30]. Additionally, hydrolysis, the critical initial step in PLA anaerobic biodegradation, is also temperature-dependent, with elevated temperatures accelerating ester bond cleavage, naturally making PLA more susceptible to degradation under thermophilic conditions [34]. Particle size is another key factor influencing PLA degradation kinetics. Several studies have demonstrated that smaller particle sizes significantly enhance anaerobic biodegradation due to their increased surface area [35,36,37]. Reducing the particle size of PLA to the 100–250 μm range solely improved the anaerobic biodegradation rate by up to 405% compared to larger particle sizes [38]. Similarly, Benn and Zitomer (2018) reported that reducing the particle size of PLA from 2 mm to 0.5 mm increased the anaerobic biodegradation rate by 50% under thermophilic conditions [36]. This enhanced biodegradation of smaller PLA particles is primarily attributed to the increased surface area-to-volume ratio, which increases the polymer’s surface availability to the surrounding microbial environment [35]. Greater surface availability facilitates more effective microbial colonization and interaction, promoting the secretion of key extracellular enzymes such as PLA depolymerases, lipases, and esterases, which are critical for polymer breakdown [36]. Additionally, smaller particle sizes shorten the diffusion pathways for water, enzymes, and hydrolytic agents, allowing more rapid and uniform penetration into the polymer [37]. This enhanced accessibility accelerates hydrolysis, leading to the faster generation of lower-molecular-weight oligomers and monomers that can be readily assimilated by anaerobic microbial consortia [35]. In addition to particle size effects, the ISR also plays a crucial role in PLA degradation. Higher ISRs enhance biodegradation kinetics by increasing the microbial density and buffering capacity, with an ISR between 2.85 and 4 g VS/g VS recommended for optimal methane production from PLA [39]. 

Laboratory-scale findings on the AD of PLA have also been validated at the full scale. Notably, Cucina et al. (2022) [26] compared the anaerobic biodegradation performance of PLA-based cutlery under laboratory-scale and full-scale conditions. This full-scale study demonstrated promising results, where the observed degradation rate aligns well with findings from the prior laboratory-scale experiments. The full-scale process yielded a methane output of 397 ± 8 NL CH_4_ per kg of VS, translating to approximately 70.8% biodegradation. This consistency strengthens the feasibility of implementing large-scale AD strategies for PLA-based bioplastics. However, it is important to recognize that recent studies are focusing on blending PLA with other bioplastics such as PBAT to overcome the inherent brittleness of PLA. While these blends improve mechanical durability, they significantly complicate the anaerobic degradation behaviour and reduce the overall biodegradability compared to pure PLA systems [40]. Overall, PLA exhibits poor anaerobic biodegradability under mesophilic conditions but shows substantially improved degradation rates and biogas yields under thermophilic regimes, particularly when combined with particle size reduction and inoculum optimization strategies.

### 2.2. Polyhydroxy Butyrate (PHB)

PHB is another widely utilized bioplastic known for its biocompatibility, making it suitable for various applications, including medical devices and drug delivery systems. Although PHB is degradable via AD, it is crucial to understand the parameters that influence the efficiency of degradation. Thus, important parameters focusing on the suitability of PHB for AD are reviewed. In terms of temperature, PHB has been reported to exhibit degradational ability at both mesophilic and thermophilic temperature ranges. A study reported that under mesophilic conditions, PHB from different manufacturers underwent 64.3 ± 0.6% and 80.1 ± 1.8% biodegradation in approximately 25–50 days [36]. In a related study, PHBH, a copolymer of PHB and hydroxyhexanoate, demonstrated that PHBH bottles underwent significant disintegration under anaerobic conditions, reaching 97.3% disintegration after 8 weeks under mesophilic conditions [21]. Interestingly, thermophilic conditions were associated with a slight decrease in degradation kinetics and a longer lag phase compared to mesophilic digestion. This reduction in performance is likely due to faster hydrolysis and acidogenesis at elevated temperatures, leading to the accumulation of volatile fatty acids (VFAs). This excessive increase in the VFA levels may acidify the digester environment, inhibit methanogenic archaea, and halt methane production, thereby reducing the overall PHB degradation efficiency [27]. This observation aligns with findings from Nachod et al. (2021) [41], who reported that during the AD of PHBH under thermophilic conditions, VFA removal was only 53.4%, whereas under mesophilic conditions, VFA removal reached 79.6%. The enhanced VFA consumption under mesophilic digestion promoted more stable methane production, suggesting that mesophilic operation offers a more balanced degradation pathway for PHB and its copolymers. 

Further, optimizing the ISR is crucial for maximizing the methane yield from the biodegradation of PHB. A study evaluated ISRs ranging from 1 to 10 and found that ISRs between 2 and 10 achieved similar methane production levels (84.8% to 93.8%) within 10–25 days after a 6-day lag phase, whereas an ISR of 1 resulted in no methane production, indicating an insufficient inoculum density. An ISR in the 2–10 range suggests the rapid and near-complete conversion of PHB into methane. Although higher ISRs demonstrated a faster methane production rate, the ultimate methane yield remained statistically similar for ISRs between 2 and 10. While a minimum ISR is necessary for efficient degradation, a high ISR does not directly translate to a significant increase in the final methane yield [39]. Jin et al. (2022) [28] also observed instability in the process when an organic loading of 8 g VS/L was used. This pattern was observed with poly 3-hydroxybutyrate 4-hydroxybutyrate (P34HB) under mesophilic conditions. When adjusting the organic loading to 4 g VS/L, the process demonstrated no lag phase, reaching a biodegradation of 64.9% in approximately 15 days. These factors could critically affect AD, as it determines the balance between the microbial population and the available substrate. 

Additionally, particle size further influences PHB degradation by modulating the available surface area for microbial attachment and enzymatic hydrolysis. García-Depraect et al. (2022) [38] showed that PHB samples with smaller particle sizes (100–250 µm) reached a degradation plateau faster (35 days) than larger particles (500–1000 µm), which required up to 65 days. Similarly, poly (3-hydroxybutyrate-co-3-hydroxyvalerate (PHBV) samples followed the same trend. Larger particle sizes reduced the net total carbon released in the gas phase, confirming that a decreased surface area impedes biodegradation kinetics. Overall, PHB demonstrates strong potential for anaerobic degradation under both mesophilic and thermophilic conditions, with mesophilic digestion offering greater process stability due to more effective VFA management. Despite promising results, further research is needed to optimize these parameters and fully realize the large-scale AD potential of PHB.

### 2.3. Polybutylene Adipate Terephthalate (PBAT)

PBAT is an aliphatic-aromatic copolyester that has gained attention as a potential substitute for conventional plastics. Due to its favorable mechanical properties and flexibility, PBAT is frequently blended with other bioplastics like PLA to overcome limitations such as durability [40]. However, the biodegradation of PBAT under AD conditions is questionable. Several studies have reported a biodegradation rate for PBAT of around 2% under mesophilic conditions [38,42]. In addition, Jin et al. (2022) noted only a 3% loss of initial mass after 60 days of treatment [28], while another study reported a mere 13.4% degradation over 500 days [27]. Thermal analysis further confirmed PBAT’s structural resilience, with Lee et al. (2024) showing that PBAT maintained its physical integrity post-digestion, as evidenced by differential scanning calorimetry (DSC) profiles [43].

The limited biodegradability of PBAT persists even when blended with other biodegradable polymers. A study investigated PBAT/PLA-based biopolymer bags under mesophilic and thermophilic AD and found no significant differences in methane and carbon dioxide production between the PBAT/PLA blend and control setups [44]. This suggested that the PBAT/PLA materials did not contribute to biogas production under either temperature regime. Visual observations corroborated these findings, as polymer films remained intact under mesophilic conditions, with only minor colour changes detected under thermophilic digestion, indicating that the PBAT/PLA material’s high thermal stability, with melting points of 110–120 °C [44]. This resistance to degradation, while beneficial for mechanical durability, presents challenges for EoL treatment via AD. Co-digestion of the PBAT/PLA blend with food waste resulted in no significant changes in methane or carbon dioxide production compared to food waste alone, indicating that the polymers did not contribute to anaerobic biodegradation [44]. Visual assessments showed intact polymer films under mesophilic conditions, with only slight discoloration observed under thermophilic digestion [44]. A similar pattern was observed in another study, with no significant degradation observed under mesophilic or thermophilic conditions for PBAT/PLA blends [45].

Interestingly, blending PBAT with starch has been explored as a strategy to improve biodegradability. On assessing the blend of PBAT/PLA/Starch at varying percentages (10%, 20%, 30%, 40%, and 50%) in co-digestion with food waste, 30% of the bioplastic blend yielded the highest methane production, with 21.09 mL/g VS added and 23.40 mL/g VS added for mesophilic and thermophilic conditions, respectively. However, no significant difference was found between the two temperature conditions, and the individual contribution of PBAT remained uncertain [46]. To address this, the same authors conducted a subsequent study evaluating the individual contribution of each bioplastic, revealing that the methane production rate followed the following order: PBAT/PLA/Starch > PLA > PBAT, with PBAT exhibiting the lowest contribution [22]. Interestingly, a study by Álvarez-Méndez et al. (2024) on commercial PLA/PBAT bioplastic bags revealed minimal biodegradation under mesophilic conditions, with the weight loss recorded to be between 1.79 and 24.61%, demonstrating significant discrepancies between compostability certification EN 13432 and the actual AD performance [47].

These findings indicate that, beyond incubation temperature, degradation during AD may also be influenced by factors such as the particle size and material form (film or powder), and other parameters [45]. However, no significant biodegradation was observed for either PBAT pellets or powders after 40 days under mesophilic and thermophilic conditions, ruling out particle size as a contributing factor [28]. Alternative strategies, such as gas purging during digestion, also showed no enhancement, with methane production from PBAT remaining equivalent to that of the control [43]. The potential reason for PBAT’s resilience to AD could be the presence of aromatic monomers, such as terephthalic acid, which increases polymer hydrophobicity, rigidity, and crystallinity, thereby resisting enzymatic hydrolysis [48,49]. This was assessed by a novel approach that tracked the assimilation of labelled carbon from its constituent individual monomers during thermophilic digestion. The study employed isotopically labelled monomers (^13^C-adipic acid, ^13^C-butanediol, ^13^C-terephthalate) to trace the labelled carbon and understand its metabolism. Batch reactors were set up with the labelled monomers as the sole carbon source, demonstrating the microbial community’s capability to assimilate carbon from two of the three PBAT monomers—adipic acid and 1,4-butanediol; meanwhile, assimilation from terephthalate was markedly lower, corresponding to reduced methane production and suggesting a potential inhibitory effect. Further, the proteogenomic analysis identified a total of 122 labelled peptides of interest, with a significantly higher abundance observed for incubations with 1,4-butanediol. This study highlights the efficient microbial assimilation of PBAT when hydrolyzed to its monomers, emphasizing the importance of pretreatment strategies (as will be discussed in detail later) to improve PBAT biodegradability in AD [50]. Overall, PBAT exhibits high resistance to AD due to its aromatic content and structural rigidity, with minimal improvement observed through operational adjustments. Pretreatment strategies targeting polymer hydrolysis, along with future studies on co-digestion and sustainable approaches, are essential to enhance its anaerobic biodegradability.

### 2.4. Poly(Ɛ-Caprolactone) (PCL)

PCL is a synthetic aliphatic polyester produced by the polymerization of ε-caprolactone. Due to its biodegradability, biocompatibility, and relatively low melting point (around 60 °C), it has been widely investigated for various biomedical and packaging applications [51]. Given its widespread popularity and extensive commercial use, developing effective EoL management strategies for its disposal is crucial. However, studies specifically targeting PCL biodegradation under AD conditions remain relatively limited.

With respect to temperature, PCL typically exhibits only limited biodegradability under mesophilic conditions. For instance, Jin et al. (2022) [28] reported the minimal degradation (< 3%) of powdered PCL after 60 days of mesophilic AD. In contrast, under thermophilic conditions, near-complete degradation (92.3%) was achieved within the same period, with a cumulative methane production of 684.4 mL CH_4_/g VS added. Morphological analysis further confirmed active biodegradation, with surface erosion and hole formation observed on PCL particles. However, contrasting results have also been reported. Cazaudehore et al. (2023) observed 49.4 ± 0.9% biodegradation under mesophilic conditions over a prolonged 500-day period, but only minimal biogas production and limited physical alterations under thermophilic conditions [27]. These discrepancies highlight the variability across studies and the need for further investigations of the influence of operational parameters. Nevertheless, other studies have consistently reported the high mineralization of PCL (87–92%) within 45–127 days under thermophilic digestion [28,35,37]. The reported enhancement of PCL biodegradation under thermophilic conditions is attributed to its relatively low melting point (~60 °C), which facilitates polymer chain scission, disrupts crystalline regions and increases microbial accessibility, thereby promoting enzymatic hydrolysis and microbial colonization [27].

Further, blending PCL with other biodegradable polymers has shown promise in improving methane yields. Under thermophilic AD conditions, blends such as PHB-PCL (60/40) and PCL-TPS (70/30) exhibited 18% and 37% higher methane yields, respectively, compared to PCL alone, suggesting the potential advantage of co-digesting PCL with other biodegradable substrates [37]. Additionally, particle size also significantly affects the anaerobic degradation kinetics of PCL. Studies have demonstrated that powdered PCL, due to its higher surface area-to-volume ratio, achieved 92.3% biodegradation within 60 days under thermophilic conditions, whereas PCL pellets required 120 days to reach a comparable degradation level (93.5%). The lag phase was also substantially shorter for powdered PCL (11 days) compared to pellets (42 days), indicating that a reduced particle size enhances microbial colonization and accelerates hydrolysis [28].

Although thermophilic conditions favor AD of PCL, recent findings suggest that oxygen availability may be an equally critical factor influencing its degradation pathways. García-Depraect et al. (2022) reported a biodegradation rate of 77.6 ± 2.4% for PCL under mesophilic aerobic conditions, compared to only 4.5 ± 0.3% under mesophilic anaerobic conditions. The authors proposed that the hydrolysis product of PCL, 6-hydroxyhexanoic acid, requires oxygen for further metabolism via β-oxidation [38,52]. In the absence of oxygen, this intermediate likely accumulates, limiting further biodegradation. Overall, while thermophilic AD shows significant promise for PCL biodegradation, variability across studies highlights the need for further investigations of the microbial communities and metabolic pathways involved. Understanding these dynamics is essential for optimizing conditions that promote the efficient anaerobic valorization of PCL-based wastes.

### 2.5. Polybutylene Succinate (PBS)

PBS, an aliphatic polyester derived from renewable resources such as succinic acid and 1,4-butanediol, exhibits desirable properties like biodegradability, thermal stability, and mechanical strength. Given these characteristics, PBS has gained interest as a potential alternative to conventional plastics in AD systems. However, a significant knowledge gap remains regarding its anaerobic biodegradability and methane production efficiency. Several studies have evaluated the anaerobic degradation of PBS under different conditions. One of the studies reported that under mesophilic AD for 100 days, the methane yield reached 25.5 mL/g VS added, which is only 4.3–4.9% of the methane potential. The study also reported visible PBS particles post-digestion, indicating inefficient degradation. Under thermophilic conditions, PBS degradation improved slightly, with a methane yield of 180.2 mL/g VS added (30.2% of theoretical methane potential), but substantial residual material remained [19]. Similarly, Jin et al. (2022) observed the surface smoothing of PBS after mesophilic digestion and crack formation under thermophilic digestion. Despite these morphological changes, biodegradation remained minimal, with biodegradation degree values below 1% under both temperature regimes [28]. These findings are consistent across multiple studies. Dvorackova et al. (2015) reported the negligible degradation of PBS regardless of its form (thin films, thick foils, or powder) under both mesophilic and thermophilic conditions [53]. These findings are corroborated by several other studies, which all reported minimal to no biodegradation of PBS in anaerobic environments [28,35,38].

Due to the poor biodegradability of PBS, the approach of blending PBS with readily degradable polymers or additives was explored. For instance, Gadaleta et al. (2024) studied PBS–gelatin blends (80/20) under mesophilic digestion for 32 days. Initial degradation was observed, likely due to the gelatin component; however, degradation plateaued after approximately 10 days, suggesting that the microbial community rapidly exhausted the gelatin while PBS remained recalcitrant [29]. As PBS failed to degrade in most of the tested conditions, pretreatment strategies were employed. The use of NaOH at concentrations of 1–3% for a 24 h pretreatment period was explored; however, this approach yielded only minimal methane production, suggesting its ineffectiveness in enhancing PBS biodegradability [17]. Interestingly, Akimoto et al. (2024) employed a high temperature (160 °C) and pressure for the 12 h pretreatment, resulting in the complete decomposition of PBS. Subsequent hydrolysis yielded substantial biogas production, reaching 460 mL/g COD added, highlighting the promise of aggressive pretreatment strategies for overcoming PBS’s anaerobic recalcitrance [54]. Overall, PBS exhibits poor anaerobic biodegradability under both mesophilic and thermophilic conditions. Aggressive pretreatment strategies such as thermal and hydrothermal processing show potential for enhancing their degradation and methane yield, warranting further exploration.

### 2.6. Starch-Based Bioplastics

Starch-based bioplastics have emerged as a prominent category of biodegradable materials, exhibiting substantial degradability under AD conditions. These materials typically contain a mixture of starch and synthetic polyesters, as identified by Fourier Transform Infrared (FTIR) analysis [55,56]. The compositional heterogeneity significantly influences their degradation behaviour, with the starch component (constituting 20–60% by weight) undergoing preferential and rapid degradation, followed by a slower decomposition of the polyester fraction. Recent investigations have elucidated the degradation kinetics of starch-based bioplastics under different operating conditions. Sorino et al. (2024) examined starch-based shoppers (SBS) composed of 60% starch and 40% PBAT, observing an initial rapid degradation phase attributed to starch consumption, succeeded by a slower PBAT degradation phase [57]. Regarding temperature, starch-based bioplastics demonstrated compatibility with both mesophilic and thermophilic AD processes. Cazaudehore et al. (2023) reported that thermoplastic starch (TPS) achieved 82.6 ± 7.8% biodegradation under mesophilic conditions (38 °C) and 80.2 ± 4.5% under thermophilic conditions (58 °C) within 25–30 days [27]. Jin et al. (2022) corroborated these findings, showing high biodegradability for TPS under both mesophilic and thermophilic conditions. Nevertheless, the kinetics were observed to be faster at thermophilic temperatures [28]. The impact of particle size on anaerobic digestibility appears minimal but not negligible. Jin et al. (2022) noted slightly higher cumulative methane production and biodegradation rates for powdered TPS compared to pelletized forms, attributing this difference to an increased surface area for microbial colonization. After 40 days of AD, pelletized and powdered TPS achieved 47.6% and 57.9% biodegradation under mesophilic conditions, respectively, with marginally higher rates observed with powdered forms, likely due to an increased surface area for microbial attack [28]. Further, investigations into commercial starch-based products have provided insights into real-world applicability. Paola Bracciale et al. (2024) evaluated Mater-Bi products under thermophilic conditions, reporting 40–60% biodegradation for starch-based cups. Their findings suggest that particle size influences the degradation rate but not the ultimate extent of biodegradation [58]. Moreover, an interesting approach involving the integration of AD with composting has shown potential for enhancing the overall biodegradation of starch-based bioplastics. Sorino et al. (2024) demonstrated that a combined approach of 28 days of thermophilic AD followed by composting resulted in the 85% biodegradation of SBS materials. These studies collectively demonstrate that starch-based bioplastics are generally compatible with AD, with higher temperatures and smaller particle sizes promoting faster degradation [57]. Overall, starch-based bioplastics exhibit high compatibility with AD, with higher temperatures and smaller particle sizes promoting faster degradation. However, the presence of less biodegradable polyester components may limit the overall degradation extent. Integrating AD with composting presents a promising strategy for achieving comprehensive biodegradation of these materials.

### 2.7. Cellulose-Based Bioplastics

Cellulose-based bioplastics represent a class of renewable, biodegradable polymers with promising potential for integration into AD systems. Their biodegradability is influenced by several operational factors, particularly temperature, which plays a critical role in modulating degradation kinetics and methane yields. Zaborowska et al. (2023) [19] observed that thermophilic conditions (55 °C) significantly enhanced the degradation rate and extent of cellulose-based materials compared to mesophilic digestion (37 °C). Under thermophilic AD, cellulose-based substrates became visually undetectable within 2 days, with approximately 90% of the total methane production occurring within 3–4 days. The methane production ranged from 403 to 415 mL/g VS added, with experimental yields reaching 75–77% of the theoretical methane production, indicating high anaerobic degradability. However, contrasting results were presented in another study, where cellulose diacetate produced around 326.3 mL/g VS added, representing around 70.9% biodegradability under mesophilic conditions, while no significant degradation was observed at thermophilic conditions [28]. Similarly, in another study, cellulose acetate exhibited high biodegradability, with weight loss reaching 98.56% after 32 days of mesophilic AD. The ultimate methane yield achieved was 519.32 NmL CH_4_/g VS added, comparable to that of food waste substrates. The degradation trend of cellulose-based bioplastic differed from typical organic substrates, showing an initial high methane yield followed by a slower, constant production phase [59]. Extending the analysis to semi-continuous AD systems, Kosheleva et al. (2023) [59] observed the moderate degradation of cellulose acetate, with the average weight loss ranging from 40 to 45% across different operational stages. On co-digesting with food waste, the methane produced by reactors containing cellulose acetate increased over time, particularly after day 65, suggesting a delayed onset of significant cellulose acetate degradation. This delay may be attributed to the preferential degradation of more readily biodegradable co-substrates like food waste. In a related study under mesophilic and solid-state AD (TS > 30%), the co-digestion of cellulose acetate with food waste yielded 519.3 and 574.8 mL CH_4_/g VS added, respectively (Gadaleta et al., 2023 [60]). Despite the high methane yields, residual cellulose acetate in the compost lowered its quality, limiting its use to floriculture and increasing the compost rejection rates.

Interestingly, the pretreatment of cellulose-based materials prior to AD showed mixed effectiveness. While pretreatment marginally improved the degradation kinetics under mesophilic conditions, the inherently high biodegradability of cellulose-based bioplastics limited its overall impact, making the effect less pronounced compared to other bioplastic types [19]. Overall, cellulose-based materials have generally shown higher biodegradability in AD compared to other biodegradable bioplastic types such as PLA, demonstrating good potential for AD [19]. Their rapid degradation kinetics and high methane yields make them promising candidates for integration into existing organic waste management systems. However, further research is needed to optimize operational parameters and assess the long-term impacts of cellulose-based bioplastic degradation products on AD systems.

**Figure 3:**
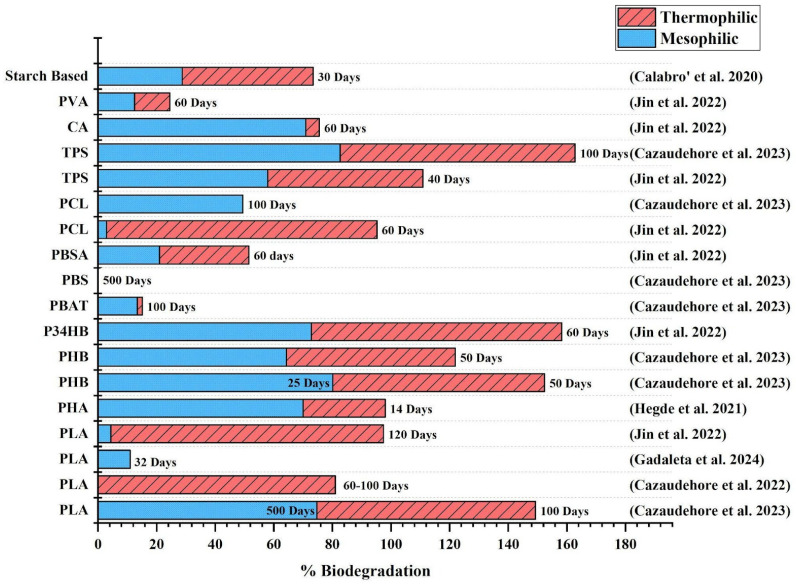
Biodegradability of common bioplastics under mesophilic and thermophilic conditions [27,28,29,31,61] [The data presented in this graph is restricted to studies that compare biodegradability under both mesophilic and thermophilic conditions, allowing for direct comparison].

In conclusion, the anaerobic biodegradability of bioplastics is highly variable and depends on multiple factors, including the polymer composition, temperature, particle size, and digestion mode. As illustrated in Figure 3, bioplastics such as PLA, PHB, starch-based polymers, and cellulose acetate exhibit relatively high biodegradability under thermophilic conditions, with degradation rates further enhanced by the reduced particle size. Conversely, bioplastics like PBAT and PBS remain recalcitrant to anaerobic degradation, even when exposed to elevated temperatures or finer particle forms. PCL shows contrasting outcomes, with some studies reporting near-complete degradation under thermophilic AD, while others indicate minimal biodegradation. These discrepancies underscore the complexity of AD processes and the influence of operational parameters on the microbial assimilation of different polymers. While PLA and PHB have been extensively studied, there remains a significant knowledge gap. Future research should focus on key areas such as (i) evaluating commercial bioplastic products as opposed to pure polymers and (ii) understanding the impact of manufacturing additives such as plasticizers or nanoparticles, polymer blending ratios, and the processing conditions affecting biodegradability in real-world applications. These findings reinforce the need for targeted enhancement strategies, such as substrate pretreatment to improve the performance of AD and co-digestion with organic substrates to digest poorly degradable bioplastics. The following section explores these approaches in detail, focusing on their potential to optimize bioplastic degradation and biogas yield in AD systems.

## 3. Enhancement Strategies

The anaerobic biodegradation of bioplastics presents a substantial challenge, primarily due to the complex chemical structures of various polymers and their limited bioavailability. These limitations are further influenced by operational parameters and the composition of microbial communities in AD systems. Various strategies have been explored to address this challenge and enhance the biodegradability of bioplastics in AD systems. These strategies include pretreatment methods to modify the physical and chemical structure of bioplastics, increasing their susceptibility to microbial attack [62], and co-digestion with other organic waste streams to create synergistic effects and enhance biogas production [63]. The following sections discuss pretreatment and co-digestion strategies in detail, drawing on recent studies that highlight their effectiveness in enhancing bioplastic degradation. Together, these strategies offer a pathway to overcoming current limitations in AD performance, facilitating improved biogas recovery and enabling the broader integration of bioplastics into circular waste management systems [64].

### 3.1. Pretreatment Techniques

Pretreatment techniques aim to overcome barriers such as crystallinity, hydrophobicity, and poor enzymatic accessibility by modifying the surface properties, physical structure, and chemical composition of bioplastics [63]. These interventions often act by introducing polar functional groups, disrupt polymer chains, and alter mechanical integrity to increase surface polarity, collectively enhancing interactions with microbial consortia and hydrolytic enzymes. Such modifications improve polymer bioavailability, thereby accelerating degradation rates and boosting biogas yields in AD [65]. A wide range of pretreatment methods have been investigated, including physical (e.g., mechanical, thermal), chemical (e.g., alkali, acid), and biological (e.g., enzymatic hydrolysis) approaches, as well as integrated combinations of these strategies, which have been discussed below

#### 3.1.1. Physical Treatment

Physical pretreatment techniques have gained attention as an effective strategy to enhance the anaerobic digestibility of recalcitrant bioplastics. These approaches aim to increase microbial accessibility by altering the surface area, crystallinity, and internal structure of the polymer matrix. This, in turn, facilitates microbial colonization and enzymatic access to the bioplastic substrate, ultimately accelerating the critical hydrolysis phase [66,67]. However, the efficacy of these pretreatments exhibits heterogeneity across the spectrum of bioplastics. The influence of physical pretreatment on the AD performance of various bioplastics drawing from all recent studies has been comprehensively summarized in Table 1.

As depicted in Table 1, physical pretreatment demonstrates varying effectiveness across bioplastic types. While the mechanical size reduction enhanced the degradation kinetics, it had only a minimal impact on the ultimate methane yield. In contrast, thermal and hydrothermal pretreatment significantly improved solubilization, leading to a substantial increase in the biogas production. Among the evaluated bioplastics, PLA and PHA showed the most favorable response, while PBAT and PBS, though generally resistant, showed a marked improvement under hydrothermal pretreatment. These general trends are corroborated by specific studies. For instance, Cazaudehore et al. (2022) investigated the impact of centrifugal milling on PLA, generating particle size fractions ranging from 1660 μm to 272 μm. While the crystallinity remained relatively unchanged (26.6–28.9%), the methane production rates were significantly enhanced for smaller particles, with 272 μm particles achieving a 7.22% increase in methane yield compared to untreated PLA. This improvement is attributed to the greater surface area and faster release of soluble additives, facilitating microbial attachment and enzymatic activity [14,16]. A clear correlation between particle size and the biodegradation yield was observed in the early stages (31 and 100 days). However, after 520 days, the ultimate methane yields converged across all particle sizes after 520 days, suggesting that mechanical treatment primarily accelerates degradation without significantly affecting the ultimate methane potential (Table 1) [16]. This phenomenon was corroborated by García-Depraect et al. (2022) for PHB and PHBV (100–1000 μm), and by Ryan et al. (2017) for PHBV particles (10–3900 μm) [38,72]. Ryan et al. further noted that smaller particles (10 μm) reached the ultimate methane potential (≈600 NL CH_4_ kg/VS added) faster (25 days) than larger ones (3900 μm, 40 days). Interestingly, it was observed that an excessive size reduction (<0.8 mm) led to VFA accumulation, temporarily halting methanogenesis. Interestingly, excessively fine grinding (<0.8 mm) was associated with VFA accumulation, which temporarily inhibited methanogenesis due to excessive acidification [72,73]. These findings collectively support the hypothesis that mechanical pretreatment improves biodegradation kinetics by increasing the microbial contact surface but does not necessarily enhance the total biogas yield. Given this, future research should also consider the trade-off between energy input for particle size reduction and its actual benefits in enhancing methane production, to identify optimal pretreatment strategies for practical applications.

In addition to mechanical processing, thermal pretreatment has been extensively investigated to improve the solubilization and biodegradability of resistant polymers like PLA. Cazaudehore et al. (2022) showed that exposing PLA to temperatures of 120 °C to 150 °C for 6–24 h significantly improved solubilization, achieving values as high as 65%, and increased methane production to 389–460 NL CH_4_/kg VS added, up from only 14 NL CH_4_/kg VS added in untreated PLA (Table 1). These high solubilization levels were confirmed by the release of acidic substances, mainly lactic acid, during the thermal pretreatment process, as evidenced by the low pH values. Interestingly, even lower pretreatment temperatures of 70 °C and 90 °C facilitated moderate PLA solubilization when combined with longer residence times of 24 and 48 h. This suggests that prolonged exposure to thermal conditions, even at relatively lower temperatures, can disrupt the PLA structure and enhance its solubilization [16]. In contrast, a previous study by Mu et al. (2021) reported a lower PLA solubilization of only 14.9% at 120 °C for shorter pretreatment durations of 10–120 min, highlighting the importance of prolonged exposure times. However, higher solubilization levels of 91.9% and 96.2% were observed at elevated temperatures of 200 °C and 240 °C, respectively [74]. The authors emphasized that both the temperature and residence time are critical parameters, as insufficient exposure yielded poor solubilization [74], while higher temperatures (200–240 °C) resulted in near-complete depolymerization.

Building upon this, an alternative form of thermal processing, digestate-mediated thermal pretreatment, was evaluated by Cucina et al. (2023), targeting starch-based shoppers and PLA cutlery. Exposure to 90 °C for 72 h improved the methane production by approximately 30%, which is hypothesized to be the readily degradable starch component [70,75]. This hypothesis was further supported by FT-IR analysis and the water uptake capacity of the pretreated SBS, which revealed that the pretreatment induced structural modifications and increased amorphousness, increasing the hydrophilicity and potential biodegradability of the polyester fraction [76]. Meanwhile, in the case of PLA cutlery, although substantial material dissolution was observed post-treatment, the FTIR spectra did not exhibit significant changes. However, a notable pH decline suggested the release of lactic acid monomers, indicative of polymer breakdown [70]. Although minimal structural alterations were detected spectroscopically, the pretreatment significantly accelerated the PLA degradation kinetics (Table 1), likely due to increased solubilization enhancing the microbial accessibility during AD [70]. The authors suggest that the observed increase in kinetic constants and reduction in the time needed to achieve an 80% methane yield suggest the possibility of reducing the hydraulic retention time (HRT) in anaerobic digesters without compromising the methane yield [70].

Further advancing the scope of thermal processing, recent studies have investigated the effectiveness of hydrothermal pretreatment (HTP) in enhancing methane production from various bioplastics, particularly those resistant to degradation under mesophilic conditions such as PLA, PBAT, PBS, and PHA [17,77]. HTP, conducted at temperatures ranging from 100 to 150 °C for durations of 1–3 h, revealed that HTP at 150 °C for 3 h significantly enhanced the solubilization efficiencies of PLA (>99%), PHA (42–43%), and PBS (58–61%). Conversely, PBAT exhibited negligible solubilization, with efficiencies between 2.5 and 2.6%. Prolonging the pretreatment duration from 1 to 3 h notably improved solubilization, particularly for PLA at 125 °C, where the solubilization efficiency increased 13–21-fold [17]. Remarkably, this increase in solubilization substantially reduced the lag phase in AD, with PLA and PHA initiating methane production immediately post-HTP at 150 °C, while PBAT and PBS showed delayed methane generation beginning after 4–7 days. With respect to the methane production yields, PLA and PHA achieved over 95% of their theoretical maximum methane yield at 150 °C. Interestingly, despite the poor solubilization of PBAT, the pretreated PBAT at 150 °C achieved a methane yield of 170 L CH_4_/kg VS added, equivalent to 30% of its theoretical value, suggesting the positive effect of hydrothermal treatment on the transformation of recalcitrant polymers into biodegradable intermediates that promoted methane production from byproducts. This suggests that complete solubilization may not be required, as physical alterations to the polymer matrix can enhance microbial accessibility and subsequent degradation [17]. This study demonstrated a strong correlation between methane yield and increasing HTP temperatures (as shown in Table 1), and represents a pioneering effort in enabling effective methane production from recalcitrant PBAT and PBS, thereby reinforcing the potential of HTP as a promising strategy for enhancing AD performance [17]. Supporting this, Ferrentino et al. (2025) investigated a more operationally realistic scenario using unground PLA spoons hydrothermally treated at 160–200 °C for 1 h. Biomethane yields of 387–490 mL CH_4_/g VS added (mesophilic) and 414–498 mL CH_4_/g VS added (thermophilic) were reported, corresponding to a 1067% and 376% increase, respectively, over untreated PLA [18]. Moreover, Marchelli et al. (2024) demonstrated that HTP could depolymerize PLA and PBS into lactic acid and succinic acid, respectively, both valuable industrial platform chemicals, which further supports HTP as a dual-purpose strategy: a chemical pretreatment for monomer recovery and a biological facilitator for enhanced digestibility. HTP may also contribute to the selective separation of biodegradable polymers from conventional plastics, improving the upstream sorting efficiency [77]. HTP may also contribute to the selective separation of biodegradable polymers from conventional plastics, improving the upstream sorting efficiency. However, these benefits were not universally observed across all feedstock combinations. Shao et al. (2025) reported that co-digesting PLA/PBAT blends with food waste under HTP offered limited benefits, largely due to (i) the formation of recalcitrant Maillard reaction products at temperatures ≥ 120 °C during food waste pretreatment, and (ii) the agglomeration and recrystallization of plastic surfaces, which inhibited microbial colonization and enzymatic access [69]. Together, these findings underscore the promise of HTP in enhancing the anaerobic degradability and methane yield of otherwise recalcitrant bioplastics. However, scalability and integration into municipal organic waste treatment infrastructure warrant further validation, particularly using realistic mixed-waste feedstocks.

Expanding beyond conventional thermal and mechanical approaches, Liu et al. (2023) [71] introduced a novel strategy by coupling hydrothermal pretreatment with a microbial electrolysis cell-assisted AD system (MEC-AD). This integrated system aimed not only to improve PLA biodegradability but also to optimize methanogenesis pathways and reduce the digestion time. In the MEC-AD setup, carbon felt and stainless-steel mesh served as the anode and cathode, respectively, connected to a direct current power supply. Hydrothermally solubilized PLA was inoculated into the system, and the microbial community was pre-adapted over several cycles prior to the experiment. The efficiency of the MEC-assisted AD system was evaluated at various applied voltages (0–0.7 V), with 0.3 V yielding optimal results. At this voltage, cumulative methane production reached 440 mL/g VS added (94% of the theoretical potential) in a much shorter period (18 days), with an 8% improvement over the AD-only control (400 mL/g VS added in 30 days). This enhancement was attributed to accelerated substrate catalysis, with no signs of process inhibition or intermediate accumulation. This improvement aligns with previous findings suggesting that MECs promote hydrogenotrophic methanogenesis by supplying electrochemically generated hydrogen as a direct electron donor for hydrogenotrophic methanogens [78,79]. Thus, the MEC-AD system not only altered methane production pathways but also shortened the required HRT while enhancing organic matter removal, leading to higher methane yields in a shorter duration compared to the conventional AD process [71].

In summary, physical pretreatment methods, especially hydrothermal processing, have been proven effective in improving the anaerobic biodegradability of recalcitrant bioplastics by enhancing solubilization and microbial accessibility. While the results vary across polymers, these techniques significantly improve degradation kinetics and methane yields. Future efforts should focus on integrating physical treatments with other strategies and validating their scalability through real-world trials. Further, novel physical pretreatment methods, such as ultrasonic irradiation and microwave irradiation, which have shown promising results in treating food waste [80,81], could be potential avenues for enhancing the anaerobic digestibility of bioplastics. 

#### 3.1.2. Chemical Treatment

Chemical pretreatment represents a widely explored strategy for improving the AD of bioplastics, particularly those exhibiting high crystallinity, hydrophobicity, or resistance to microbial hydrolysis [63,82]. These approaches typically involve exposing bioplastics to alkaline or acidic agents, which break ester or glycosidic linkages within the polymer matrix, thereby enhancing solubilization, reducing the molecular weight, and modifying surface properties [82]. This breakdown of polymer chains increases the surface area, resulting in more accessible sites for microbial attachment and enzyme activity, improvising the biodegradation process [25]. Additionally, chemical pretreatments can modify the surface properties of bioplastics, making them more hydrophilic, thus increasing the accessibility and biodegradability of the pretreated bioplastic material, leading to higher biogas yields and faster degradation rates [62]. Recent studies have highlighted the differential effects of acid and base pretreatments across bioplastic types, which are comprehensively tabulated in Table 2.

As mentioned in Table 2, chemical pretreatments, particularly alkaline treatments, showed significant potential for enhancing methane yield. However, the response was highly polymer-specific. Additionally, it was observed that an excessive alkali concentration could lead to process inhibition or negative impacts. In particular, Battista et al. (2021) [83] evaluated sugarcane cellulosic plates (SCCP), starch-based materials, and PLA under acidic (pH 2—HCl) and alkaline (pH 12—NaOH) conditions for 48 h. Contrary to expectations, basic pretreatment reduced methane yields by 12–20% across SCCP, starch cutlery, and PLA, while acidic pretreatment had no statistically significant impact. The authors suggested that these outcomes may be due to mesophilic operational temperatures (35 °C), where hydrolytic activity is less responsive to pH-induced polymer breakdown. Such a study highlights the complexity of pretreatment effects on different bioplastics and the importance of considering factors such as temperature and potential inhibitory effects when optimizing AD processes. Although previous research suggests limited success with NaOH pretreatment for PLA biodegradation under mesophilic conditions [83], Hobbs et al. (2019) demonstrated that the extended alkaline pretreatment of PLA using 10 M NaOH for 15 days at 21 °C substantially improved solubilization and increased the methane yield by 35%, achieving 928.18 mL CH_4_/g VS by day 70. Notably, the lag phase was eliminated entirely, underscoring the influence of treatment duration and concentration on PLA digestibility [86]. This finding highlights the potential of extended alkaline pretreatment and its influence on AD efficiency, particularly for materials typically considered less biodegradable under specific temperatures. Consistent with this study, the impact of NaOH pretreatment (7 days—37 °C) on the AD of various bioplastics, including PLA, PLA/PCL (80/20), PHB, PHBH, and PHBV, was investigated and a significant reduction in the lag phase for pre-treated PLA compared to the untreated control was observed, with biogas production initiated immediately after inoculation, a contrast to the extended lag times seen in untreated controls [84]. Notably, pretreatment enabled the AD of PLA and PLA/PCL, which were recalcitrant under the tested conditions without pretreatment [84].

Interestingly, the response to chemical pretreatment varies significantly by polymer type. The PHBH displayed the lowest susceptibility to pretreatment (80% solubilization), and all PHBs exhibited significantly extended lag phases (19.9–23.7 days) following pretreatment compared to untreated controls. While PHB and PHBV showed minimal change in methane yield after NaOH treatment, PHBH experienced a reduction of 14%, suggesting potential inhibitory effects or incomplete hydrolysis. Additionally, the proportion of carbon converted to methane remained consistent across all materials, while the proportion converted to carbon dioxide was significantly reduced compared to untreated bioplastic digestion [84].

In contrast, Benn and Zitomer (2018) reported that the NaOH treatment of PHB at pH 12 for 24 h significantly improved biodegradability, reducing the lag time and increasing BMP compared to neutral pH conditions. They also observed manufacturer-specific variations, likely due to differences in the plasticizer content and synthesis methods, emphasizing the need for tailored pretreatment strategies [36]. Extending the effect of NaOH across other bioplastics, researchers systematically investigated the impact of NaOH pretreatment on a broader range of bioplastics [85]. Their approach involved applying NaOH solutions at different concentrations (1%, 3%, and 5%) for 24 h at 25 °C. The materials exhibited varying responses: PBSA, TPS, PLA, and PPC displayed high solubility, while PBS showed partial dissolution. Notably, PBAT and PCL demonstrated strong resistance to the pretreatment. In terms of biodegradability, cellulose diacetate (CDA), P34HB, PBS, PBSA, PCL, and PLA showed improvement at appropriate concentrations (Table 2) [85]. TPS biodegradability showed a slight decrease, while PPC and PBAT were unaffected. However, PBS remained relatively resistant to degradation [85], consistent with Zaborowska et al. (2023), who reported that PBS pretreated with 0.1 M KOH at 35 °C for 2 h exhibited only minor increases in methane yield, with visible polymer fragments still present post-AD [19].

In summary, chemical pretreatment, particularly alkaline hydrolysis, emerges as a potent method for enhancing the anaerobic digestibility of a wide spectrum of bioplastics. Across numerous studies, NaOH-based treatments demonstrated significant improvements in solubilization, degradation kinetics, and methane yields, especially for recalcitrant polymers such as PLA, PHBH, CDA, and certain PHAs. The underlying mechanism involves hydroxide-induced ester bond cleavage, which is strongly influenced by the chemical structure of ester substituents (R and R′) within the polymer backbone (RCOOR′), leading to a reduced molecular weight, increased hydrophilicity, and greater microbial accessibility [36,87]. Notably, several studies reported the complete elimination of lag phases, a critical advantage for improving reactor efficiency. However, the treatment outcomes varied significantly with polymer type, chemical concentration, treatment duration, and operational conditions. For instance, highly biodegradable bioplastics like starch blends or PHBH showed a reduced performance, potentially due to acidification during rapid hydrolysis. Conversely, PBAT and PBS remained largely resistant, even at elevated NaOH concentrations [84]. In contrast, acidic pretreatments (e.g., HCl) generally showed limited efficacy, with several studies reporting negligible improvements or even reductions in methane yield [83]. These findings suggest that acidic hydrolysis is less effective for disrupting the polymer structure of most bioplastics under mesophilic conditions, likely due to incomplete chain scission or the formation of inhibitory byproducts. Thus, while alkaline pretreatment shows broader applicability, acidic methods may require coupling with thermal or enzymatic strategies to enhance effectiveness.

Nevertheless, both approaches carry practical limitations. Alkaline pretreatments risk introducing sodium toxicity, disrupting microbial balance, and reducing methane conversion efficiency when not carefully optimized [83,85,88]. Moreover, chemical pretreatment processes are often energy-intensive and involve hazardous reagents, raising environmental and safety concerns [89]. Future efforts should prioritize thermo-alkaline hybrid strategies, explore greener or bio-based chemical alternatives, and incorporate techno-economic assessments to ensure industrial scalability and alignment with sustainability goals.

#### 3.1.3. Physicochemical Treatment

Building upon the promising results from individual physical and chemical strategies, recent studies have explored synergistic physicochemical approaches to enhance the anaerobic digestibility of recalcitrant bioplastics. Thermo-alkaline and mechano-alkaline combinations are the most commonly applied, where physical processes such as milling or thermal treatment increase surface area and disrupt the polymer structure, while chemical agents (primarily alkalis) promote hydrolysis and solubilization [61,90]. This integration facilitates better microbial access, reduces lag phases, and can substantially improve methane yields [86]. Table 3 provides a summary of the effects of physicochemical pretreatment on various bioplastics.

This table reinforces the ability of physicochemical treatments, particularly at temperatures above 70 °C and pH > 10, to significantly improve methane yields. Across studies, thermal alkaline pretreatment consistently emerged as a promising strategy for enhancing biodegradability, particularly for polymers that are otherwise recalcitrant under standard AD conditions. Benn and Zitomer (2018) tested it on PHB and PLA, applying NaOH at pH 10–12 and temperatures ranging from 35 to 90 °C for 3–48 h. PHB pretreated at 55 °C and pH 12 for 24 h showed a 13% increase in methane yield, while PLA, typically non-biodegradable under mesophilic conditions, achieved 86 NmL CH_4_/g ThOD at 90 °C, pH 10–12, for 48 h. Lag phases were reduced by 60% for PHB and up to 98% for PLA, demonstrating the effectiveness of thermo-alkaline pretreatment for enhancing biodegradability [36] (Table 3). Nie et al. (2024) investigated the effects of thermal–alkaline pretreatment (1% NaOH, 70 °C, 48 h) on PBAT pellets, PLA pellets, PBAT/PLA plastic bags, and PBAT/PLA/Starch plastic bags. Under mesophilic conditions, no significant difference in methane production was observed between pretreated and untreated samples, except for starch-containing blends, likely due to the hydrolysable starch fraction [93]. This contrasts with Benn and Zitomer (2018), as PLA and PBAT did not show appreciable methane conversion despite pretreatment during the 100-day incubation [36,93]. In contrast, under thermophilic conditions, pretreatment accelerated early methane production, though the cumulative yields remained similar to the untreated controls [93] (Table 3). In a similar effort, Venkiteshwaran et al. (2019) applied thermal–alkaline pretreatment (55 °C, pH 12, 24 h) along with particle size reduction (<0.15 mm) to PHB. This resulted in a shorter lag time (3–5 days), with a 4.4–6.8% increase in methane yield and a ~50% reduction in acclimation time, demonstrating added benefits during co-digestion scenarios [91]. Ashraf Joolaei et al. (2024) [92] subjected PLA to thermo-alkaline pretreatment using 6 N KOH (50–200 g OH^−^/kg BP) at 60–100 °C for 6–10 h. Solubilization increased with treatment intensity (15–40%). The conditions and the methane yield, along with its effect, are provided in Table 3. As untreated PLA was non-biodegradable under mesophilic conditions, methane yield improvements were assessed relative to the weakest pretreatment. Remarkably, strong thermo-alkaline pretreatment (40% solubilization) achieved a methane conversion efficiency of 91.4%, surpassing solubilization rates and contradicting the trends observed in prior studies, where higher solubilization did not always translate to improved methane yields [16,84]. This could have potentially been due to using harsher pretreatment conditions (higher alkaline dosage, temperature, and reaction time), which might have released recalcitrant that cannot be converted into methane. FTIR analysis by Ashraf Joolaei et al. (2024) further confirmed bond disruption via increased carboxylate (COO^−^ at 1595 cm^−1^) and hydroxyl (–OH at 3430–3510 cm^−1^) signals, indicative of early-stage PLA degradation [92]. Cazaudehore et al. (2022) adopted a similar thermo-alkaline strategy, substituting NaOH/KOH with Ca(OH)_2_, and reported a significant enhancement in PLA solubilization, particularly at lower temperatures (70–90 °C) [16]. Methane yields were comparable across multiple conditions, 1 h at 150 °C, 6 h at 120 °C, 24 h at 90 °C, and 48 h at 70 °, with 2.5% *w*/*v* Ca(OH)_2_ proving sufficient for substantial degradation. Increasing the concentration to 5% *w*/*v* showed no additional benefit at both 70 and 90 °C, indicating a performance plateau. Thermo-alkaline pretreatment also resulted in visible surface erosion, increased porosity, and numerous holes on the PLA granules’ surface, which can be attributed to the breakdown of polymer chains during pretreatment. Considering industrial implementation, pretreatment at 70 °C with 2.5% *w*/*v* Ca(OH)_2_ appears to be the most appropriate condition, which resulted in the 73% bioconversion of PLA in 30 days under mesophilic conditions [16]. Additionally, the choice of Ca(OH)_2_ is advantageous for industrial applications due to its lower cost, greater availability, and lower corrosiveness compared to NaOH, offering economic and operational benefits in large-scale AD setups [94,95].

In conclusion, physicochemical pretreatment strategies, particularly thermo- and mechano-alkaline approaches, hold strong potential for enhancing the AD of bioplastics by synergistically improving solubilization, reducing the lag phase and enhancing the methane yield. However, these methods come with drawbacks like increased energy consumption, the potential formation of inhibitory compounds, and additional chemical usage, impacting cost and sustainability. Nevertheless, the physicochemical pretreatment strategies can potentially serve as a critical bridge between bench-scale feasibility and scalable AD optimization for bioplastic waste valorization. Therefore, to ensure their practical relevance, future efforts must focus on optimizing process conditions to balance treatment efficiency with energy and chemical inputs.

#### 3.1.4. Enzymatic Treatment

Enzymatic pretreatment strategically employs specific enzymes to catalyze the hydrolysis of bioplastic polymer chains into smaller, more bioavailable molecules. The selection of the enzyme requires consideration of the specific bioplastic type and its chemical composition, the enzyme concentration, the substrate concentration, and the reaction time. For example, proteases and lipases have demonstrated effectiveness in degrading PLA [96], while cutinases are frequently utilized for PBAT hydrolysis [97]. Unlike physico-chemical methods, enzymatic treatments operate under milder conditions, reducing the formation of inhibitory byproducts [98]. While only a limited number of studies have been conducted in this domain, the potential for further exploration remains vast. Comprehensive data on enzymatic and thermal-enzymatic pretreatments for improving the anaerobic degradability of bioplastic blends are shown in Table 4.

The collective findings suggest that enzyme-based strategies can substantially enhance methane production, particularly when applied in combination with thermal treatment. The degree of improvement, however, was highly dependent on the enzyme type, substrate composition, and operational conditions. While enzymatic treatments demonstrated notable enhancements, certain combinations yielded marginal or even negative effects, underscoring the importance of enzyme–substrate specificity and pre-treatment optimization. Jiang et al. (2023) [99] showed that the enzymatic pretreatment of PLA/PBAT/starch blends using amylase, lipase, and proteinase K (0.1 g/L, 72 h) significantly enhanced methane yields, especially with proteinase K, exhibiting increased efficiency at both mesophilic and thermophilic operating conditions. Additionally, the degradation time of the bioplastic bags witnessed a noticeable reduction from 30 days to 24 days. Further, SEM analysis revealed substantial surface erosion, while FTIR analysis revealed a weakening of the characteristic peak at 1700 cm^−1^, which corresponds to the carbonyl group of ester bonds present in both PLA and PBAT. A novel enzymatic modification strategy was developed to enhance the anaerobic co-digestion of PLA/PBAT blends by embedding enzymes such as proteinase K (PK), porcine pancreatic lipase (PPL), and amylase (Amy) within the polymer matrix (Liu et al., 2024 [100]). PLA and PBAT were dissolved in a dichloromethane–water solution and coated with enzyme solution during film formation, resulting in bioplastics containing 1% enzyme by weight. Fluorescence microscopy confirmed stable enzyme incorporation. Upon AD, the modified plastic, especially with PK, achieved a fivefold increase in biodegradability (5.21% to 29.70%), reduced the methane production cycle from 24 to 20 days and increased CMY (Table 4). Kinetic modelling indicated that the embedded enzymes accelerated hydrolysis by facilitating bioplastic breakdown and simultaneously enhancing the degradation of co-digested food waste through the cleavage of resistant chemical bonds.

In conclusion, enzymatic pretreatment offers a targeted, mild, and environmentally friendly strategy to improve the anaerobic digestibility of bioplastics, particularly blends like PLA/PBAT and starch-based composites. However, practical challenges remain. Its widespread application is limited by challenges such as enzyme specificity, operational stability, and high production costs. Although the recombinant expression of target enzymes could aid in bulk production [101], enzyme activity is susceptible to deactivation under AD conditions. This requires the maintenance of optimal pH and temperature (pH 4–12.5; 35–65 °C) for proteinase K, (pH 5—12; 30–60 °C) lipase and (pH 4.5–9; 23–58 °C) amylase, as well as possible replenishment to maintain efficacy [20,100]. Economic feasibility also poses a key constraint, as enzyme production and purification remain costly, particularly for large-scale applications [102]. Nevertheless, the substantial gains in methane production and degradation kinetics justify continued research and optimization. Future work should focus on broadening the scope of identifying enzymes with substrate specificity, improving enzyme stability, and evaluating synergistic integration with thermal or microbial augmentation to enhance the scalability and cost-effectiveness of enzymatic pretreatment strategies for bioplastic waste valorization.

### 3.2. Co-Digestion

**Figure 4:**
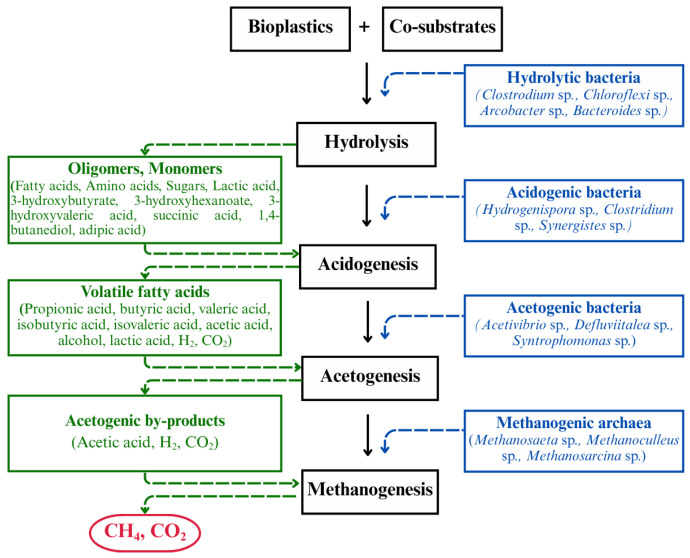
Schematic representation of microbially mediated bioplastic degradation under anaerobic co-digestion (only the representative genus and by-products are presented as illustrative examples among those commonly observed in AD systems).

While pretreatment strategies offer substantial improvements in the hydrolysis and solubilization of recalcitrant bioplastics, co-digestion serves as a complementary approach. As bioplastics typically lack nitrogen and other essential nutrients, this can lead to an imbalance in the C/N ratio when digested alone. Thereby, co-digestion presents a practical and synergistic strategy, where co-substrates primarily serve as a carbon and nitrogen source. In such a case, where bioplastics are processed alongside nutrient-rich co-substrates such as food waste, manure, and sewage sludge, it not only compensates for these deficiencies but also enables optimal microbial functioning and process stability, and allows for the use of existing AD infrastructure, reducing the need for additional capital investment [14]. Optimizing the C/N ratio is a key benefit of co-digestion. Ideal C/N ratios for AD typically range between 20:1 and 30:1. Ratios exceeding 30:1 can lead to nitrogen limitation and reduced methanogenic activity, while lower ratios (<20:1) may result in ammonia accumulation, which is inhibitory to methanogens [103]. Beyond nutrient balancing, co-digestion promotes the development of diverse and robust microbial consortia with broader enzymatic capacities, enabling the degradation of complex organic compounds [104]. The presence of readily degradable co-substrates also provides accessible carbon sources that stimulate microbial proliferation and the production of hydrolytic enzymes. This enzymatic hydrolysis initiates a sequential microbial process that leads to the bioconversion of bioplastic-derived carbon into CH_4_ and CO_2_, thereby accelerating the degradation of more recalcitrant materials like bioplastics [105]. The overall microbial degradation pathway of bioplastics under anaerobic co-digestion is illustrated in Figure 4, showing the key microbial groups and their associated metabolic products across each AD stage. Furthermore, co-substrates contribute to the buffering capacity, mitigating the effects of pH fluctuations and organic loading shocks. This is especially beneficial in bioplastic digestion, where process instability is often a limiting factor [36,106]. The effectiveness of co-digestion in enhancing bioplastic biodegradation depends significantly on the type and characteristics of the co-substrate used. The following section reviews recent findings regarding both substrate selection and the optimization of key AD parameters that aid in efficient co-digestion strategies for bioplastics.

#### 3.2.1. Selection of Co-Substrates

The selection of appropriate co-substrates is crucial for effective bioplastic co-digestion. Given the nutrient limitations of bioplastics, combining them with nutrient-dense, readily biodegradable substrates can significantly enhance anaerobic degradation. Suitable co-substrates stimulate microbial growth, support enzymatic activity, and facilitate more balanced digestion conditions, ultimately improving both bioplastic breakdown and biogas yield. Among the most widely studied co-substrates are food waste and sewage sludge, both of which offer distinct advantages in terms of their nutrient content, buffering capacity, and compatibility with existing AD systems [14,107].

(i) Food Waste: Numerous studies have demonstrated the synergistic effects of co-digesting bioplastics with food waste. Yu et al. (2023) observed a 28.4% increase in CMP when co-digesting PBAT/PLA/starch bioplastics with food waste under thermophilic conditions, along with improved bioplastic conversion efficiencies ranging from 9.11 to 11.2% [22]. Similarly, Hobbs et al. (2019) reported enhanced methane production and PLA degradation when co-digested with food waste, particularly when combined with alkaline pretreatment [86]. Kang et al. (2022) further corroborated these findings by demonstrating methane yield improvements of 8.5–26.6% for PLA and 12.7–25.5% for PHA during co-digestion with food waste compared to mono-digestion [108]. Additionally, pilot-scale experiments by Maragkaki et al. (2023) confirmed the feasibility of integrating bioplastics like PLA derived from food waste into existing digester systems, reporting an 8% increase in biomethane production without operational disruptions, signifying the potential for seamless integration [109]. These findings highlight the effectiveness of food waste as a co-substrate, suggesting that co-digestion not only optimizes biogas production but also enhances bioplastic degradation by supplying readily accessible nutrients, stimulating microbial proliferation, and inducing the production of hydrolytic enzymes involved in polymer breakdown.
(ii) Sewage Sludge: Another common nutrient-rich co-substrate, high in nitrogen and phosphorus, has emerged as a highly effective co-substrate for enhancing bioplastic degradation in AD systems. Its favorable nutrient profile, inherent buffering capacity, and abundance make it a suitable complement to carbon-rich bioplastics that often lack essential macronutrients for microbial metabolism [23]. A study by Akimoto et al. (2024) demonstrated that co-digesting sewage sludge with PLA hydrolysate resulted in a methane yield of 414 L/kg COD added, highlighting the stable conversion potential of bioplastic components in the presence of sludge [54]. Similarly, Cazaudehore et al. (2023) explored the co-digestion of PLA or PHB with biowastes, reporting not only increased reactor stability but also an improved bioplastic degradation efficiency. Notably, they observed a remarkable 103% conversion of PHB to methane, while pretreated PLA achieved a 77.5% conversion rate, suggesting the potential for near-complete bioplastic degradation under optimized co-digestion conditions [110]. Further insights were provided by García-Depraect et al. (2024) [21], who evaluated PHBH co-digestion (20% VS basis) with various substrates, including municipal sludge, food waste, or swine manure; this showed promising results, with methane yields comparable to or slightly higher than those of organic wastes alone. In the case of municipal sludge, the addition of PHBH increased the methane production by around 16%. These findings highlight the potential for integrating PHBH bioplastics into existing waste management systems, offering the dual benefit of bioplastic disposal and enhanced biogas production. However, the study also emphasized that bioplastic degradability varied significantly with the type of co-substrate and inoculum used. Complementing these findings, Shafana Farveen et al. (2025) evaluated the co-digestion of PHBH with sewage sludge under batch and semi-batch conditions, reporting a stable volumetric methane production rate of 281.17 ± 22.48 NmL CH_4_/L-d during continuous PHBH feeding over 93 days [111]. Additional support for sewage sludge as an effective co-substrate comes from Pangallo et al. (2023) [23], who combined it with the organic fraction of municipal solid waste (OFMSW) and Mater-Bi bioplastics. Their results showed a near doubling of the methane yield and a significantly improved process stability compared to mono-digestion. These findings suggest that sewage sludge not only enhances degradation through nutrient supplementation but also favors the establishment of the syntrophic bacterial–archaea consortia essential for polymer breakdown, VFA conversion, and methanogenesis [105]. These studies collectively underscore the effectiveness of sewage sludge as a co-substrate, particularly in the co-digestion of PHBH, PLA, and PHB. The consistent improvements in methane yield, reactor stability, and bioplastic conversion highlight its suitability for integration into existing municipal and industrial AD infrastructure.

In summary, both food waste and sewage sludge have demonstrated strong potential as co-substrates for enhancing the AD of bioplastics. Their high biodegradability and nutrient richness help overcome the compositional limitations of carbon-rich bioplastics, improving microbial activity, enzymatic hydrolysis, and biogas yields. The observed process stability in these systems can be attributed to two key mechanisms, one being pH buffering, which helps maintain optimal pH levels within the digester. This is crucial for the activity of methanogenic bacteria, which are sensitive to pH fluctuations [91]. The latter is the dilution of inhibitors, where the potential inhibitory compounds generated via bioplastic breakdown are diluted during co-digestion, mitigating their negative impact on microbial activity and overall process stability [112]. However, these benefits must be balanced against operational challenges such as ammonia inhibition, especially when using nitrogen-rich substrates like sewage sludge. Excess ammonia can suppress methanogenesis, reducing biogas productivity. Therefore, the successful implementation of co-digestion strategies requires the careful selection of co-substrates along with the optimization of key process parameters, including ISR, the organic loading rate (OLR), and pH, to ensure process efficiency and stability [113].

#### 3.2.2. Optimization of Key Parameter

(i) The inoculum-to-substrate ratio (ISR) represents the mass ratio of active microbial biomass to total substrate, directly influencing the biokinetic balance between nutrient availability and microbial activity within the digester. The theoretical basis for ISR optimization stems from Monod kinetics, where the microbial growth rate depends on both the substrate concentration and biomass concentration [114]. Yu et al. (2023) [46] investigated the influence of ISR on methane production during the co-digestion of bioplastic bags with food waste, identifying that a 30% bioplastic-to-food waste ratio (by weight) is optimal for achieving peak methane production. This finding suggests that a balanced ratio between the carbon-rich bioplastics and the nutrient-rich food waste creates a favorable environment for microbial populations to achieve robust hydrolysis and minimize acid accumulation. A suboptimal ISR may lead to process acidification, leading to reduced methanogenic efficiency.
(ii) The organic loading rate (OLR) determines the amount of organic matter fed into the digester per unit of digester volume per unit of time. Maintaining an appropriate OLR is crucial to prevent the digester from being overloaded, acid accumulation, and microbial inhibition. The optimization of OLR depends on the balancing of substrate addition with the microbial capacity to degrade it, following the first-order kinetics [115]. In a co-digestion study involving sewage sludge, OFMSW, and bioplastics, Pangallo et al. (2023) successfully increased the OLR from 1 g VS/L-d to 2 g VS/L-d without compromising the methane output. This demonstrated that, with an appropriate balance, adequate buffering, and microbial acclimation, well-managed co-digestion systems can handle higher organic loads, enhancing biogas productivity [23].
(iii) Temperature is a key factor influencing microbial metabolism and bioplastic degradation rates. Comparative studies have shown that thermophilic digestion (~55 °C) often outperforms mesophilic conditions (~37 °C) in terms of methane yield. Nachod et al. (2021) reported that PHBV treatments under thermophilic conditions produced 271 mL CH_4_/g VS over 104 days, outperforming mesophilic setups [41]. Similarly, Yu et al. (2023) found that thermophilic digestion reduced the lag phase for methane production [46]. While thermophilic conditions often lead to higher yields, the ideal temperature can be influenced by the specific polymer type (Figure 3).
(iv) Maintaining pH within the optimal range of 6.5–8.0 is critical for stable AD. Deviations outside this range can inhibit methanogens, especially during bioplastic degradation, which may release acidic intermediates. Cioabla et al. (2012) emphasized the importance of continuous pH monitoring and buffering, particularly as certain bioplastics can lead to the accumulation of acidic degradation products (VFAs) that can negatively impact the process [116,117].

By optimizing these key parameters, co-digestion systems can be optimized to accelerate bioplastic degradation, enhance methane yield, and maintain reactor stability. Furthermore, Navaneethan et al. (2011) demonstrated the potential for increased electricity generation in municipal wastewater treatment plants through the co-digestion of biodegradable plastics, emphasizing the broader energy recovery potential of these strategies [118].

## 4. Conclusions

The global transition towards bioplastics necessitates efficient and sustainable waste management strategies, with AD emerging as a promising pathway for both energy recovery and environmental protection. However, the recalcitrant nature of many bioplastics under conventional AD conditions presents major challenges. This review critically evaluated enhancement strategies, such as co-digestion and a range of pre-treatment approaches including physical, chemical, physicochemical, and enzymatic methods, that aim to overcome these limitations by improving hydrolysis, biodegradability, and methane yield. Co-digestion with substrates like food waste and sludge improves the C/N balance and microbial synergy, while pretreatments facilitate polymer disruption, solubilization, and accelerated degradation. While research has largely concentrated on PLA and PHB, expanding investigations to less-explored polymers like PBS and PBAT is critical for achieving comprehensive and scalable solutions for bioplastic valorization via AD. Future efforts should focus on standardizing operating parameters, developing hybrid and energy-efficient technologies, and conducting long-term pilot trials alongside techno-economic and life-cycle assessments. Additionally, the success of bioplastic valorization depends not only on process optimization but also on the implementation of effective waste collection and segregation systems. Policy frameworks should mandate and promote infrastructure investments that enable the integration of bioplastic waste into the existing AD and organic waste treatment facilities. Together, these advances can pave the way for incorporating bioplastics into circular bioeconomy frameworks, aligning with the environmental sustainability goals of renewable energy generation.

## Figures and Tables

**Table 1:** Effect of physical pretreatments on AD of various common bioplastics.

Bioplastic	AD Condition	Pretreatment	Methane Yield	Effect	Reference
PLA	38 °C—520 days	-	429 ± 21 NL CH_4_/kg VS added		[16]
		Mechanical Grinding—1660 μm	427 ± 9 NL CH_4_/kg VS added	0.47% ↓	
		Mechanical Grinding—1140 μm	441 ± 10 NL CH_4_/kg VS added	2.80% ↑	
		Mechanical Grinding—808 μm	441 ± 17 NL CH_4_/kg VS added	2.80% ↑	
		Mechanical Grinding—502 μm	455 ± 7 NL CH_4_/kg VS added	6.06% ↑	
		Mechanical Grinding—272 μm	460 ± 11 NL CH_4_/kg VS added	7.22% ↑	
PLA	38 °C—25 days	-	14 ± 4 NL CH_4_/kg VS added		[16]
		Thermal 150 °C—6 h	389 ± 20 NL CH_4_/kg VS added	100+ % ↑	
		Thermal 120 °C—24 h	370 ± 11 NL CH_4_/kg VS added	100+ % ↑	
PLA	38° C—32 days	-	9.45 L CH_4_/kg VS added		[17]
		Hydrothermal 100 °C—2% *w*/*w* BP—3 h	45.73 L CH_4_/kg VS added	100+ % ↑	
		Hydrothermal 125 °C—2% *w*/*w* BP—3 h	455.98 L CH_4_/kg VS added	100+ % ↑	
		Hydrothermal 150 °C—2% *w*/*w* BP—3 h	454.99 L CH_4_/kg VS added	100+ % ↑	
PHA	38 °C—32 days	-	430.45 L CH_4_/kg VS added		[17]
		Hydrothermal 100 °C—2% *w*/*w* BP—3 h	511.24 NL CH_4_/kg VS added	18.7% ↑	
		Hydrothermal 125 °C—2% *w*/*w* BP—3 h	523.54 NL CH_4_/kg VS added	21.6% ↑	
		Hydrothermal 150 °C—2% *w*/*w* BP—3 h	534.20 NL CH_4_/kg VS added	24.1% ↑	
PBAT	38 °C—32 days	-	NA		[17]
		Hydrothermal 100 °C—2% *w*/*w* BP—3 h	NA		
		Hydrothermal 125 °C—2% *w*/*w* BP—3 h	31.88 NL CH_4_/kg VS added	100+ % ↑	
		Hydrothermal 150 °C—2% *w*/*w* BP—3 h	174.27 NL CH_4_/kg VS added	100+ % ↑	
PBS	38 °C—32 days	-	NA		[17]
		Hydrothermal 100 °C—2% *w*/*w* BP—3 h	69.43 NL CH_4_/kg VS added	100+ % ↑	
		Hydrothermal 125 °C—2% *w*/*w* BP—3 h	171.09 NL CH_4_/kg VS added	100+ % ↑	
		Hydrothermal 150 °C—2% *w*/*w* BP—3 h	492.37 NL CH_4_/kg VS added	100+ % ↑	
Starch-based	55 °C—30 days	-	320.9 NL CH_4_/kg VS added		[68]
		Hydrothermal 134 °C—20 min	439.4 NL CH_4_/kg VS added	36.9% ↑	
PLA	55 °C—30 days	-	360.3 L CH_4_/kg VS added		[68]
		Hydrothermal 134 °C—20 min	426.4 L CH_4_/kg VS added	18.3% ↑	
PLA/PBAT blend (30 μm)	26 days	-	94.65 L CH_4_/kg VS added	-	[69]
		Hydrothermal 80 °C—10.7% *w*/*w* BP—1 h	116.75 L CH_4_/kg VS added	23.35% ↑	
		Hydrothermal 100 °C—10.7% *w*/*w* BP—1 h	110.79 mLCH4/g VS	17.95% ↑	
		Hydrothermal 120 °C—10.7% *w*/*w* BP—1 h	92.47 L CH_4_/kg VS added	2.30% ↓	
PLA/PBAT blend (40 μm)	26 days	-	98.61 L CH_4_/kg VS added	-	[69]
		Hydrothermal 80 °C—10.7% *w*/*w* BP—1 h	98.69 L CH_4_/kg VS added	0.08% ↑	
		Hydrothermal 100 °C—10.7% *w*/*w* BP—1 h	104.42 L CH_4_/kg VS added	5.89% ↑	
		Hydrothermal 120 °C—10.7% *w*/*w* BP—1 h	92.47 L CH_4_/kg VS added	6.23% ↓	
Starch-based	55 °C—60 days	-	63 ± 8 NL CH_4_/kg TS added		[70]
		Digestate mediated Thermal 90 °C—1.25% *w*/*w* BP—72 h	82 ± 4 NL CH_4_/kg TS added	30% ↑	
PLA	55 °C—60 days	-	138 ± 3 NL CH_4_/kg TS added		[70]
		Digestate mediated Thermal 90 °C—1.25% *w*/*w* BP—72 h	139 ± 9 NL CH_4_/kg TS added	0.7% ↑	
PLA	35 °C—30 days	-	400 mL/g VS		[71]
	35 °C—18 days	Microbial Electrolysis Cell Assisted AD + Hydrothermal 180 °C—2 h—0.3V	440 mL/g VS	10% ↑	

Upward arrows indicate the percentage increase relative to the control, while downward arrows represent the corresponding percentage decrease. 100+ indicates an improvement of more than 100% relative to the baseline value.

**Table 2:** Effect of chemical pretreatments on AD of various common bioplastics.

Bioplastic	AD Condition	Pretreatment	Methane Yield	Effect	Reference
PHB	35 °C—40 days	-	233 NmL CH_4_/g ThOD ^a^		[36]
		Alkaline 35 °C—pH 7 NaOH—48 h	359 NmL CH_4_/g ThOD ^a^	54% ↑	
PHB	35 °C—40 days	-	199 NmL CH_4_/g ThOD ^a^		[36]
		Alkaline 35 °C—pH 12 NaOH- 24 h	398 NmL CH_4_/g ThOD ^a^	100% ↑	
Sugar Cane Cellulosic Fibre (SCCP) Plates	35 °C—250 days	-	391.14 ± 21.06 LCH_4_/kg VS		[83]
		Acidic pH 2—1N HCl—48 h	342.64 ± 23.76 L CH_4_/kg VS	12% ↓	
		Alkaline pH 12—1N NaOH—48 h	339.90 ± 38.15 L CH_4_/kg VS	13% ↓	
Starch-Based Bags—UNI EN13342	35 °C—250 days	-	200.91 ± 4.60 L CH_4_/kg VS		[83]
		Acidic pH 2—1N HCl—48 h	203.87 ± 3.19 L CH_4_/kg VS	1% ↑	
		Alkaline pH 12—1N NaOH—48 h	158.05 ± 4.51 L CH_4_/kg VS	21% ↓	
Starch Based Cutleries	35 °C—250 days	-	312.50 ± 8.20 L CH_4_/kg VS		[83]
		Acidic pH 2—1N HCl—48 h	302.51 ± 6.64 L CH_4_/kg VS	3% ↓	
		Alkaline pH 12—1N NaOH—48 h	252.87 ± 7.90 L CH_4_/kg VS	19% ↓	
PLA	35 °C—250 days	-	130.00 ± 6.70 L CH_4_/kg VS		[83]
		Acidic pH 2—1N HCl—48 h	125.29 ± 5.40 L CH_4_/kg VS	3% ↓	
		Alkaline pH 12—1N NaOH—48 h	103.93 ± 2.51 L CH_4_/kg VS	20% ↓	
PHB	37 °C—80 days	-	432.7 ± 6.7 NmL CH_4_/g VS		[84]
		Alkaline 37 °C—2M NaOH—7 days	426.7 ± 2.1 NmL CH_4_/g VS	1% ↓	
PHBH	37 °C—80 days	-	462.3 ± 5.5 NmL CH_4_/g VS		[84]
		Alkaline 37 °C—2M NaOH—7 days	397.0 ± 15.6 NmL CH_4_/g VS	14% ↓	
PHBV	37 °C—80 days	-	435.1 ± 15 NmL CH_4_/g VS		[84]
		Alkaline 37 °C—2M NaOH—7 days	437.2 ± 8.3 NmL CH_4_/g VS	0.4% ↓	
PLA	37 °C—80 days	-	3.1 ± 2.9 NmL CH_4_/g VS		[84]
		Alkaline 37 °C—1M NaOH—7 days	361.0 ± 1.8 NmL CH_4_/g VS	100+ % ↑	
PLA/PCL Blend	37 °C—80 days	-	7.4 ± 1.2 NmL CH_4_/g VS		[84]
		Alkaline 37 °C—1M NaOH—7 days	386 ± 6.4 NmL CH_4_/g VS	100+ % ↑	
CDA	55 °C—60 days	-	21.1 L CH_4_/kg VS		[85]
		Alkaline 25 °C—1% *w*/*w* NaOH—24 h	187.4 L CH_4_/kg VS	100+ % ↑	
		Alkaline 25 °C—3% *w*/*w* NaOH—24 h	363.6 L CH_4_/kg VS	100+ % ↑	
		Alkaline 25 °C—5% *w*/*w* NaOH—24 h	392.0 L CH_4_/kg VS	100+ % ↑	
P34HB	55 °C—60 days	-	500.3 L CH_4_/kg VS		[85]
		Alkaline 25 °C—1% *w*/*w* NaOH—24 h	561.8 L CH_4_/kg VS	12% ↑	
		Alkaline 25 °C—3% *w*/*w* NaOH—24 h	566.7 L CH_4_/kg VS	13% ↑	
		Alkaline 25 °C—5% *w*/*w* NaOH—24 h	532.4 L CH_4_/kg VS	6.4% ↑	
PBAT	55 °C—60 days	-	3.3 L CH_4_/kg VS		[85]
		Alkaline 25 °C—1% *w*/*w* NaOH—24 h	4.5 L CH_4_/kg VS	36% ↑	
		Alkaline 25 °C—3% *w*/*w* NaOH—24 h	5.4 L CH_4_/kg VS	63% ↑	
		Alkaline 25 °C—5% *w*/*w* NaOH—24 h	5.4 L CH_4_/kg VS	63% ↑	
PBS	55 °C—60 days	-	4.8 L CH_4_/kg VS		[85]
		Alkaline 25 °C—1% *w*/*w* NaOH—24 h	12.2 L CH_4_/kg VS	100+ % ↑	
		Alkaline 25 °C—3% *w*/*w* NaOH—24 h	25.3 L CH_4_/kg VS	100+ % ↑	
		Alkaline 25 °C—5% *w*/*w* NaOH—24 h	34.1 L CH_4_/kg VS	100+ % ↑	
PCL	55 °C—60 days	-	684.4 L CH_4_/kg VS		[85]
		Alkaline 25 °C—1% *w*/*w* NaOH—24 h	704.4 L CH_4_/kg VS	2.9% ↑	
		Alkaline 25 °C—3% *w*/*w* NaOH—24 h	732.5 L CH_4_/kg VS	7% ↑	
		Alkaline 25 °C—5% *w*/*w* NaOH—24 h	415.9 L CH_4_/kg VS	39% ↓	
PLA	55 °C—120 days	-	434.4 L CH_4_/kg VS		[85]
		Alkaline 25 °C—1% *w*/*w* NaOH—24 h	446.9 L CH_4_/kg VS	2.6% ↑	
		Alkaline 25 °C—3% *w*/*w* NaOH—24 h	446.3 L CH_4_/kg VS	2.7% ↑	
		Alkaline 25 °C—5% *w*/*w* NaOH—24 h	462.6 L CH_4_/kg VS	6.5% ↑	
PPC	55 °C—60 days	-	570.8 L CH_4_/kg VS		[85]
		Alkaline 25 °C—1% *w*/*w* NaOH—24 h	547.0 L CH_4_/kg VS	4.2% ↓	
		Alkaline 25 °C—3% *w*/*w* NaOH—24 h	551.1 L CH_4_/kg VS	3.5% ↓	
		Alkaline 25 °C—5% *w*/*w* NaOH—24 h	546.9 L CH_4_/kg VS	4.1% ↓	
PVA	55 °C—60 days	-	68.9 L CH_4_/kg VS		[85]
		Alkaline 25 °C—1% *w*/*w* NaOH—24 h	75.5 L CH_4_/kg VS	9.5% ↑	
		Alkaline 25 °C—3% *w*/*w* NaOH—24 h	79.1 L CH_4_/kg VS	14.7% ↑	
		Alkaline 25 °C—5% *w*/*w* NaOH—24 h	74.3 L CH_4_/kg VS	7.8% ↑	
TPS	55 °C—40 days	-	310.2 L CH_4_/kg VS		[85]
		Alkaline 25 °C—1% *w*/*w* NaOH—24 h	279.4 L CH_4_/kg VS	9.9% ↓	
		Alkaline 25 °C—3% *w*/*w* NaOH—24 h	290.5 L CH_4_/kg VS	6.3% ↓	
		Alkaline 25 °C—5% *w*/*w* NaOH—24 h	281.0 L CH_4_/kg VS	9.4% ↓	
PLA	37 °C—70 days	-	686.36 mL CH_4_/g VS ^b^		[86]
		Alkaline 21 °C—10M NaOH pH 11—15 days	928.18 mL CH_4_/g VS ^b^	35% ↑	
Cellulose-based	35 °C—100 days	-	311.4 L CH_4_/kg VS		[19]
		Alkaline 0.1M KOH—2 h	315 L CH_4_/kg VS	1.2% ↑	
PBS-based	35 °C—100 days	-	25.5 L CH_4_/kg VS		[19]
		Alkaline 0.1M KOH—2 h	29.3 L CH_4_/kg VS	14% ↑	

^a^ Calculated from provided VS and methane yield values for cross-study comparison. ^b^ Values are reported in NmL CH_4_/g ThOD, where ThOD represents the theoretical oxygen demand of the bioplastic. Upward arrows indicate the percentage increase relative to the control, while downward arrows represent the corresponding percentage decrease. 100+ indicates an improvement of more than 100% relative to the baseline value.

**Table 3:** Effect of physicochemical pretreatments on AD of various common bioplastics.

Bioplastic	AD Condition	Pretreatment	Methane Yield	Effect	Reference
PHB	35 °C—40 days	-	316 NmL CH_4_/g ThOD ^a^		[36]
		Thermal Alkaline 55 °C—pH 10 NaOH—24 h	322 NmL CH_4_/g ThOD ^a^	2% ↑	
PHB	35 °C—40 days	-	316 NmL CH_4_/g ThOD ^a^		[36]
		Thermal Alkaline 55 °C—pH 12 NaOH—24 h	357 NmL CH_4_/g ThOD ^a^	13% ↑	
PLA	35 °C—40 days	-	NA		[36]
		Thermal Alkaline 90 °C—pH 10 NaOH—48 h	86 NmL CH_4_/g ThOD ^a^	100+ %↑	
PHB	35 °C—175 days	-	88 ± 0.4 L CH_4_		[91]
		Thermal Alkaline 55 °C—pH 12—24 h	94 ± 0.7 L CH_4_	6.8% ↑	
PLA	38 °C—30 days	Thermal 90 °C—48 h	136 ± 8 NL CH_4_/kg VS		[16]
		Thermal Chemical 90 °C—0.5% Ca(OH)_2_—48 h	178 ± 11 NL CH_4_/kg VS	30% ↑	
		Thermal Chemical 90 °C—1.25% Ca(OH)_2_—48 h	260 ± 3 NL CH_4_/kg VS	91% ↑	
		Thermal Chemical 90 °C—2.5% Ca(OH)_2_—48 h	352 ± 14 NL CH_4_/kg VS	100+ % ↑	
		Thermal Chemical 90 °C—5% Ca(OH)_2_—48 h	354 ± 1 NL CH_4_/kg VS	100+ % ↑	
PLA	38 °C—30 days	Thermal 70 °C—48 h	48 ± 4 NL CH_4_/kg VS		[16]
		Thermal Chemical 70 °C—0.5% Ca(OH)_2_—48 h	167 ± 11 NL CH_4_/kg VS	100+ % ↑	
		Thermal Chemical 70 °C—1.25% Ca(OH)_2_—48 h	286 ± 14 NL CH_4_/kg VS	100+ % ↑	
		Thermal Chemical 70 °C—2.5% Ca(OH)_2_—48 h	381 ± 11 NL CH_4_/kg VS	100+ % ↑	
		Thermal Chemical 70 °C—5% Ca(OH)_2_—48 h	338 ± 41 NL CH_4_/kg VS	100+ % ↑	
PLA	55 °C—32 days	Particle Size—1–2 mm Thermal Alkaline—Weak 87.5 g OH^−^/kgBP, 70 °C—3.25 h	184.1 ± 12.3 mL CH_4_/g COD		[92]
		Particle Size—1–2 mm Thermal Alkaline—Medium 125.0 g OH^−^/kgBP, 80 °C—5.5 h	220.5 ± 7.6 mL CH_4_/g COD	20% ↑	
		Particle Size—1–2 mm Thermal Alkaline—Strong 162.5 g OH^−^/kgBP, 90 °C—7.7 h	258.2 ± 12.9 mL CH_4_/g COD	40% ↑	
PLA	55 °C—100 days	-	366 ± 17 NmL CH_4_/g VS added		[93]
		Thermal Alkaline 70 °C—1% NaOH (*w*/*v*)—48 h	334 ± 22 NmL CH_4_/g VS added	9% ↓	
PBAT	55 °C—100 days	-	91 ± 7 NmL CH_4_/g VS added		
		Thermal Alkaline 70 °C—1% NaOH (*w*/*v*)—48 h	91 ± 14 NmL CH_4_/g VS added	0%	
PLA/PBAT	55 °C—100 days	-	148 ± 18 NmL CH_4_/g VS added		
		Thermal Alkaline 70 °C—1% NaOH (*w*/*v*)—48 h	90 ± 2 NmL CH_4_/g VS added	48% ↓	
PLA/PBAT/Starch	55 °C—100 days	-	117 ± 13 NmL CH_4_/g VS added		
		Thermal Alkaline 70 °C—1% NaOH (*w*/*v*)—48 h	115 ± 5 NmL CH_4_/g VS added	1% ↓	

^a^ Values are reported in NmL CH_4_/g ThOD, where ThOD represents the theoretical oxygen demand of the bioplastic. Upward arrows indicate the percentage increase relative to the control, while downward arrows represent the corresponding percentage decrease. 100+ indicates an improvement of more than 100% relative to the baseline value.

**Table 4:** Effect of biological pretreatments on AD of various common bioplastics.

Bioplastic	AD Condition	Pretreatment	CMP	Effect	Reference
PLA/PBAT/Starch Blend	35 °C—35 days	-	61.42 mL		[99]
		Enzymatic—Amylase 35 °C—0.1g/L VS—72 h	85.73 mL	39.6% ↑	
		Enzymatic—Lipase 35 °C—0.1g/L VS—72 h	107.09 mL	74.4% ↑	
		Enzymatic—Proteinase K 35 °C—0.1g/L VS—72 h	158.90 mL	100+ % ↑	
PLA/PBAT/Starch Blend	55 °C—35 days	-	170.19 mL		[99]
		Thermal Enzymatic—Amylase 55 °C—0.1g/L VS—72 h	208.82 mL	22.6% ↑	
		Thermal Enzymatic—Lipase 55 °C—0.1g/L VS—72 h	225.52 mL	32.5% ↑	
		Thermal Enzymatic—Proteinase K 55 °C—0.1g/L VS—72 h	273.94 mL	60.9% ↑	
PLA/ PBAT Blend	37 °C—30 days	-	237.7 mL/g VS		[100]
		Enzymatic Modification With 1% Amylase Remolded at 40 °C	246.5 mL/g VS	3.7% ↑	
		Enzymatic Modification With 1% Lipase (PPL) Remolded at 40 °C	241.4 mL/g VS	1.5% ↑	
		Enzymatic Modification With 1% Proteinase K Remolded at 40 °C	234.7 mL/g VS	1.2% ↓	

Upward arrows indicate the percentage increase relative to the control, while downward arrows represent the corresponding percentage decrease. 100+ indicates an improvement of more than 100% relative to the baseline value.

## Data Availability

The original contributions presented in the study are included in the article.

## Abbreviations

The following abbreviations are used in this manuscript:

AD	Anaerobic Digestion
ISR	Inoculum-to-substrate ratio
PLA	Polylactic acid
PHB	Polyhydroxy butyrate
PBAT	Polybutylene adipate-co-terephthalate
PBS	Polybutylene succinate
PCL	Polycaprolactone
CMP	Cumulative methane production
VS	Volatile solids
PHBV	Poly(3-hydroxybutyrate-co-3-hydroxyhexanoate)
VFAs	Volatile fatty acids
BMP	Biochemical methane potential
COD	Chemical oxygen demand
ThOD	Theoretical oxygen demand

## References

[1] Williams A.T., Rangel-Buitrago N. (2022). The Past, Present, and Future of Plastic Pollution. Mar. Pollut. Bull..

[2] Salhofer S., Jandric A., Soudachanh S., Le Xuan T., Tran T.D. (2021). Plastic Recycling Practices in Vietnam and Related Hazards for Health and the Environment. Int. J. Environ. Res. Public Health.

[3] Dokl M., Copot A., Krajnc D., Van Fan Y., Vujanović A., Aviso K.B., Tan R.R., Kravanja Z., Čuček L. (2024). Global Projections of Plastic Use, End-of-Life Fate and Potential Changes in Consumption, Reduction, Recycling and Replacement with Bioplastics to 2050. Sustain. Prod. Consum..

[4] Ganguly R.K., Chakraborty S.K. (2024). Plastic Waste Management during and Post Covid19 Pandemic: Challenges and Strategies towards Circular Economy. Heliyon.

[5] Gu J.-D. (2021). Biodegradability of Plastics: The Issues, Recent Advances, and Future Perspectives. Environ. Sci. Pollut. Res. Int..

[6] Lauer N.E., Nowlin M.B. (2022). A Framework for Inland Cities to Prevent Marine Debris: A Case Study from Durham, North Carolina. Front. Mar. Sci..

[7] Peydayesh M., Bagnani M., Mezzenga R. (2021). Sustainable Bioplastics from Amyloid Fibril-Biodegradable Polymer Blends. ACS Sustain. Chem. Eng..

[8] Muthusamy M.S., Pramasivam S. (2019). Bioplastics--an Eco-Friendly Alternative to Petrochemical Plastics. Curr. World Environ..

[9] Lomwongsopon P., Varrone C. (2022). Contribution of Fermentation Technology to Building Blocks for Renewable Plastics. Fermentation.

[10] Escobar N., Haddad S., Börner J., Britz W. (2018). Land Use Mediated GHG Emissions and Spillovers from Increased Consumption of Bioplastics. Environ. Res. Lett..

[11] Lamberti F.M., Román-Ramírez L.A., Wood J. (2020). Recycling of Bioplastics: Routes and Benefits. J. Polym. Environ..

[12] Vargas-Estrada L., García-Depraect O., Zimmer J., Muñoz R. (2025). Analysis of Biological Treatment Technologies, Their Present Infrastructures and Suitability for Biodegradable Food Packaging–A Review. J. Environ. Manag..

[13] García-Depraect O., Bordel S., Lebrero R., Santos-Beneit F., Börner R.A., Börner T., Muñoz R. (2021). Inspired by Nature: Microbial Production, Degradation and Valorization of Biodegradable Bioplastics for Life-Cycle-Engineered Products. Biotechnol. Adv..

[14] Abraham A., Park H., Choi O., Sang B.-I. (2021). Anaerobic Co-Digestion of Bioplastics as a Sustainable Mode of Waste Management with Improved Energy Production—A Review. Bioresour. Technol..

[15] Quecholac-Piña X., Hernández-Berriel M.D.C., Mañón-Salas M.D.C., Espinosa-Valdemar R.M., Vázquez-Morillas A. (2020). Degradation of Plastics under Anaerobic Conditions: A Short Review. Polymers.

[16] Cazaudehore G., Guyoneaud R., Vasmara C., Greuet P., Gastaldi E., Marchetti R., Leonardi F., Turon R., Monlau F. (2022). Impact of Mechanical and Thermo-Chemical Pretreatments to Enhance Anaerobic Digestion of Poly(lactic Acid). Chemosphere.

[17] Im S., Hwang I., Weonjae K., Kim D.-H., Kang J.-H., Kang S. (2024). Enhancing Methane Production Potential of Biodegradable Plastics by Hydrothermal Pretreatment. Environ. Technol. Innov..

[18] Ferrentino R., Marchelli F., Bevilacqua A., Fiori L., Andreottola G. (2025). Hydrothermal Pre-Treatments Can Make PLA and PBS Bioplastics Suitable for Anaerobic Digestion. J. Environ. Chem. Eng..

[19] Zaborowska M., Bernat K., Pszczółkowski B., Kulikowska D., Wojnowska-Baryła I. (2023). Assessment of Biodegradability of Cellulose and Poly(butylene Succinate)-Based Bioplastics under Mesophilic and Thermophilic Anaerobic Digestion with a View towards Biorecycling. Waste Manag..

[20] Ferdeș M., Dincă M.N., Moiceanu G., Zăbavă B.Ș., Paraschiv G. (2020). Microorganisms and Enzymes Used in the Biological Pretreatment of the Substrate to Enhance Biogas Production: A Review. Sustain. Sci. Pract. Policy.

[21] García-Depraect O., Martínez-Mendoza L.J., Aragão Börner R., Zimmer J., Muñoz R. (2024). Biomethanization of Rigid Packaging Made Entirely of poly(3-Hydroxybutyrate-Co-3-Hydroxyhexanoate): Mono- and Co-Digestion Tests and Microbial Insights. Bioresour. Technol..

[22] Yu C., Dongsu B., Tao Z., Xintong J., Ming C., Siqi W., Zheng S., Yalei Z. (2023). Anaerobic Co-Digestion of Three Commercial Bio-Plastic Bags with Food Waste: Effects on Methane Production and Microbial Community Structure. Sci. Total Environ..

[23] Pangallo D., Gelsomino A., Fazzino F., Pedullà A., Calabrò P.S. (2023). The Fate of Biodegradable Plastic during the Anaerobic Co-Digestion of Excess Sludge and Organic Fraction of Municipal Solid Waste. Waste Manag..

[24] Di Bartolo A., Infurna G., Dintcheva N.T. (2021). A Review of Bioplastics and Their Adoption in the Circular Economy. Polymers.

[25] Zhang W., Heaven S., Banks C.J. (2018). Degradation of Some EN13432 Compliant Plastics in Simulated Mesophilic Anaerobic Digestion of Food Waste. Polym. Degrad. Stab..

[26] Cucina M., Carlet L., De Nisi P., Somensi C.A., Giordano A., Adani F. (2022). Degradation of Biodegradable Bioplastics under Thermophilic Anaerobic Digestion: A Full-Scale Approach. J. Clean. Prod..

[27] Cazaudehore G., Monlau F., Gassie C., Lallement A., Guyoneaud R. (2023). Active Microbial Communities during Biodegradation of Biodegradable Plastics by Mesophilic and Thermophilic Anaerobic Digestion. J. Hazard. Mater..

[28] Jin Y., Cai F., Song C., Liu G., Chen C. (2022). Degradation of Biodegradable Plastics by Anaerobic Digestion: Morphological, Micro-Structural Changes and Microbial Community Dynamics. Sci. Total Environ..

[29] Gadaleta G., De Gisi S., Picuno C., Heerenklage J., Kuchta K., Sorrentino A., Notarnicola M., Oliviero M. (2024). Assessment of Methane Production, Disintegration, and Biodegradation Potential of Bioplastic Waste in Anaerobic Digestion Systems. J. Environ. Chem. Eng..

[30] Itävaara M., Karjomaa S., Selin J.-F. (2002). Biodegradation of Polylactide in Aerobic and Anaerobic Thermophilic Conditions. Chemosphere.

[31] Hegde S., Diaz C.A., Dell E.M., Trabold T.A., Lewis C.L. (2021). Investigation of process parameters on the anaerobic digestion of a poly(hydroxyalkonate) film. Eur. Polym. J..

[32] Kolstad J.J., Vink E.T.H., De Wilde B., Debeer L. (2012). Assessment of Anaerobic Degradation of IngeoTM Polylactides under Accelerated Landfill Conditions. Polym. Degrad. Stab..

[33] Lyu S., Untereker D. (2009). Degradability of Polymers for Implantable Biomedical Devices. Int. J. Mol. Sci..

[34] Vargas L.F., Welt B.A., Teixeira A., Pullammanappallil P., Balaban M., Beatty C. (2009). Biodegradation of Treated Polylactic Acid (PLA) under Anaerobic Conditions. Trans. ASABE.

[35] Yagi H., Ninomiya F., Funabashi M., Kunioka M. (2013). Thermophilic Anaerobic Biodegradation Test and Analysis of Eubacteria Involved in Anaerobic Biodegradation of Four Specified Biodegradable Polyesters. Polym. Degrad. Stab..

[36] Benn N., Zitomer D. (2018). Pretreatment and Anaerobic Co-Digestion of Selected PHB and PLA Bioplastics. Front. Environ. Sci. Eng. China.

[37] Narancic T., Verstichel S., Reddy Chaganti S., Morales-Gamez L., Kenny S.T., De Wilde B., Babu Padamati R., O’Connor K.E. (2018). Biodegradable Plastic Blends Create New Possibilities for End-of-Life Management of Plastics but They Are Not a Panacea for Plastic Pollution. Environ. Sci. Technol..

[38] García-Depraect O., Lebrero R., Rodriguez-Vega S., Bordel S., Santos-Beneit F., Martínez-Mendoza L.J., Aragão Börner R., Börner T., Muñoz R. (2022). Biodegradation of Bioplastics under Aerobic and Anaerobic Aqueous Conditions: Kinetics, Carbon Fate and Particle Size Effect. Bioresour. Technol..

[39] Cazaudehore G., Guyoneaud R., Lallement A., Gassie C., Monlau F. (2022). Biochemical Methane Potential and Active Microbial Communities during Anaerobic Digestion of Biodegradable Plastics at Different Inoculum-Substrate Ratios. J. Environ. Manag..

[40] Jubinville D., Awad M., Lee H.-S., Mekonnen T.H. (2024). Effect of Compatibilizers on the Physico-Mechanical Properties of a Poly(lactic Acid)/ Poly(butylene Adipate-Co-Terephthalate) Matrix with Rice Straw Micro-Particle Fillers. J. Polym. Environ..

[41] Nachod B., Keller E., Hassanein A., Lansing S. (2021). Assessment of Petroleum-Based Plastic and Bioplastics Degradation Using Anaerobic Digestion. Sustain. Sci. Pract. Policy.

[42] Svoboda P., Dvorackova M., Svobodova D. (2019). Influence of Biodegradation on Crystallization of Poly (butylene Adipate-Co-Terephthalate). Polym. Adv. Technol..

[43] Lee E.S., Park S.Y., Kim C.G. (2024). Comparison of Anaerobic Digestion of Starch- and Petro-Based Bioplastic under Hydrogen-Rich Conditions. Waste Manag..

[44] Peng W., Wang Z., Shu Y., Lü F., Zhang H., Shao L., He P. (2022). Fate of a Biobased Polymer via High-Solid Anaerobic Co-Digestion with Food Waste and Following Aerobic Treatment: Insights on Changes of Polymer Physicochemical Properties and the Role of Microbial and Fungal Communities. Bioresour. Technol..

[45] Peng W., Nie R., Lü F., Zhang H., He P. (2024). Biodegradability of PBAT/PLA Coated Paper and Bioplastic Bags under Anaerobic Digestion. Waste Manag..

[46] Yu C., Dongsu B., Tao Z., Zhe K., Xintong J., Siqi W., Ming C., Zheng S., Yalei Z. (2023). Anaerobic Co-Digestion of PBAT/PLA/starch Commercial Bio-Plastic Bags with Food Waste: Effects on Methane Production and Microbial Community Structure. Biochem. Eng. J..

[47] Álvarez-Méndez S.J., Ramos-Suárez J.L., Ritter A., Mata González J., Camacho Pérez Á. (2023). Anaerobic Digestion of Commercial PLA and PBAT Biodegradable Plastic Bags: Potential Biogas Production and 1H NMR and ATR-FTIR Assessed Biodegradation. Heliyon.

[48] Zumstein M.T., Rechsteiner D., Roduner N., Perz V., Ribitsch D., Guebitz G.M., Kohler H.-P.E., McNeill K., Sander M. (2017). Enzymatic Hydrolysis of Polyester Thin Films at the Nanoscale: Effects of Polyester Structure and Enzyme Active-Site Accessibility. Environ. Sci. Technol..

[49] Jia X., Zhao K., Zhao J., Lin C., Zhang H., Chen L., Chen J., Fang Y. (2023). Degradation of Poly(butylene Adipate-Co-Terephthalate) Films by Thermobifida Fusca FXJ-1 Isolated from Compost. J. Hazard. Mater..

[50] Poulsen J.S., Trueba-Santiso A., Lema J.M., Echers S.G., Wimmer R., Nielsen J.L. (2023). Assessing Labelled Carbon Assimilation from Poly Butylene Adipate-Co-Terephthalate (PBAT) Monomers during Thermophilic Anaerobic Digestion. Bioresour. Technol..

[51] Labet M., Thielemans W. (2009). Synthesis of Polycaprolactone: A Review. Chem. Soc. Rev..

[52] Kunioka M., Ninomiya F., Funabashi M. (2009). Biodegradation of Poly(butylene Succinate) Powder in a Controlled Compost at 58 °C Evaluated by Naturally-Occurring Carbon 14 Amounts in Evolved CO_2_ Based on the ISO 14855-2 Method. Int. J. Mol. Sci..

[53] Dvorackova M., Svoboda P., Kostka L., Pekarova S. (2015). Influence of Biodegradation in Thermophilic Anaerobic Aqueous Conditions on Crystallization of Poly(butylene Succinate). Polym. Test..

[54] Akimoto S., Tsubota J., Hidaka T., Fujiwara T. (2024). Continuous Co-Digestion of Sewage Sludge and Highly Concentrated Waste Bioplastic Hydrolyzate without Shortening Hydraulic Retention Time. Waste Manag. Bull..

[55] Cucina M., De Nisi P., Trombino L., Tambone F., Adani F. (2021). Degradation of Bioplastics in Organic Waste by Mesophilic Anaerobic Digestion, Composting and Soil Incubation. Waste Manag..

[56] Ruggero F., Gori R., Lubello C. (2019). Methodologies to Assess Biodegradation of Bioplastics during Aerobic Composting and Anaerobic Digestion: A Review. Waste Manag. Res..

[57] Sorino D., Bartolucci L., Cordiner S., Costa G., Lombardi F., Mulone V. (2024). Numerical Framework for Anaerobic Digestion And/or Composting of Bioplastics and Organic Waste Performance Evaluation under Real-like Large Scale Operating Conditions. Sustain. Chem. Pharm..

[58] Paola Bracciale M., De Gioannis G., Falzarano M., Muntoni A., Polettini A., Pomi R., Rossi A., Sarasini F., Tirillò J., Zonfa T. (2024). Disposable Mater-Bi^®^ Bioplastic Tableware: Characterization and Assessment of Anaerobic Biodegradability. Fuel.

[59] Kosheleva A., Gadaleta G., De Gisi S., Heerenklage J., Picuno C., Notarnicola M., Kuchta K., Sorrentino A. (2023). Co-Digestion of Food Waste and Cellulose-Based Bioplastic: From Batch to Semi-Continuous Scale Investigation. Waste Manag..

[60] Gadaleta G., Ferrara C., De Gisi S., Notarnicola M., De Feo G. (2023). Life Cycle Assessment of End-of-Life Options for Cellulose-Based Bioplastics When Introduced into a Municipal Solid Waste Management System. Sci. Total Environ..

[61] Calabro’ P.S., Folino A., Fazzino F., Komilis D. (2020). Preliminary Evaluation of the Anaerobic Biodegradability of Three Biobased Materials Used for the Production of Disposable Plastics. J. Hazard. Mater..

[62] Shruti V.C., Kutralam-Muniasamy G. (2019). Bioplastics: Missing Link in the Era of Microplastics. Sci. Total Environ..

[63] Bátori V., Åkesson D., Zamani A., Taherzadeh M.J., Sárvári Horváth I. (2018). Anaerobic Degradation of Bioplastics: A Review. Waste Manag..

[64] Folino A., Karageorgiou A., Calabrò P.S., Komilis D. (2020). Biodegradation of Wasted Bioplastics in Natural and Industrial Environments: A Review. Sustain. Sci. Pract. Policy.

[65] Ciuffi B., Fratini E., Rosi L. (2024). Plastic Pretreatment: The Key for Efficient Enzymatic and Biodegradation Processes. Polym. Degrad. Stab..

[66] Zhu X., Zhu S., Zhao Z., Kang X., Ju F. (2023). Microbiome Dynamics during Anaerobic Digestion of Food Waste and the Genetic Potential for Poly (lactic Acid) Co-Digestion. Chem. Eng. J..

[67] Narancic T., Cerrone F., Beagan N., O’Connor K.E. (2020). Recent Advances in Bioplastics: Application and Biodegradation. Polymers.

[68] Angelini S., Gallipoli A., Montecchio D., Angelini F., Gianico A., Sbicego M., Braguglia C.M. (2025). The Strategic Role of a Mild Hydrothermal Pretreatment in Enhancing Anaerobic Degradation of Commercial Bio-Based Compostable Plastics Associated to Food Waste. J. Environ. Manag..

[69] Shao H., Yu M., Zhao L., Wang P., Meng X., Ren L. (2025). Impact of Hydrothermal Pretreatment on Enhancing Anaerobic Co-Digestion of Food Waste and Biodegradable Plastics. J. Environ. Chem. Eng..

[70] Cucina M., De Nisi P., Adani F. (2023). Thermo-Alkaline Pre-Treatment Operated by Digestate Improved Biomethane Production of Bioplastic. Bioresour. Technol. Rep..

[71] Liu W., Abrha H., Dai Y., Li J., Liu M., Maryam B., Jiao S., Zhang P., Liu X. (2023). Microbial Electrolysis Cell Assisted Anaerobic Digestion System Boosted the Methane Production from Polylactic Acid by Optimizing the Methanogenesis Pathway. Biochem. Eng. J..

[72] Ryan C.A., Billington S.L., Criddle C.S. (2017). Assessment of Models for Anaerobic Biodegradation of a Model Bioplastic: Poly(hydroxybutyrate-Co-Hydroxyvalerate). Bioresour. Technol..

[73] Barakat A., Mayer-Laigle C., Solhy A., Arancon R.A.D., de Vries H., Luque R. (2014). Mechanical Pretreatments of Lignocellulosic Biomass: Towards Facile and Environmentally Sound Technologies for Biofuels Production. RSC Adv..

[74] Mu L., Zhang L., Ma J., Zhu K., Chen C., Li A. (2021). Enhanced Biomethanization of Waste Polylactic Acid Plastic by Mild Hydrothermal Pretreatment: Taguchi Orthogonal Optimization and Kinetics Modeling. Waste Manag..

[75] Elfehri Borchani K., Carrot C., Jaziri M. (2015). Biocomposites of Alfa Fibers Dispersed in the Mater-Bi^®^ Type Bioplastic: Morphology, Mechanical and Thermal Properties. Compos. Part A Appl. Sci. Manuf..

[76] Papa G., Cucina M., Echchouki K., De Nisi P., Adani F. (2023). Anaerobic Digestion of Organic Waste Allows Recovering Energy and Enhancing the Subsequent Bioplastic Degradation in Soil. Resour. Conserv. Recycl..

[77] Marchelli F., Mattonai M., Ferrentino R., La Nasa J., Pecorelli N., Modugno F., Andreottola G., Ribechini E., Fiori L. (2024). Fostering Bioplastics Circularity through Hydrothermal Treatments: Degradation Behavior and Products. ACS Sustain. Chem. Eng..

[78] Guo X., Liu J., Xiao B. (2013). Bioelectrochemical Enhancement of Hydrogen and Methane Production from the Anaerobic Digestion of Sewage Sludge in Single-Chamber Membrane-Free Microbial Electrolysis Cells. Int. J. Hydrogen Energy.

[79] Zhao Z., Zhang Y., Woodard T.L., Nevin K.P., Lovley D.R. (2015). Enhancing Syntrophic Metabolism in up-Flow Anaerobic Sludge Blanket Reactors with Conductive Carbon Materials. Bioresour. Technol..

[80] Deepanraj B., Sivasubramanian V., Jayaraj S. (2017). Effect of Substrate Pretreatment on Biogas Production through Anaerobic Digestion of Food Waste. Int. J. Hydrogen Energy.

[81] Yue L., Cheng J., Tang S., An X., Hua J., Dong H., Zhou J. (2021). Ultrasound and Microwave Pretreatments Promote Methane Production Potential and Energy Conversion during Anaerobic Digestion of Lipid and Food Wastes. Energy.

[82] Yadav B., Pandey A., Kumar L.R., Tyagi R.D. (2020). Bioconversion of Waste (water)/residues to Bioplastics- A Circular Bioeconomy Approach. Bioresour. Technol..

[83] Battista F., Frison N., Bolzonella D. (2021). Can Bioplastics Be Treated in Conventional Anaerobic Digesters for Food Waste Treatment?. Environ. Technol. Innov..

[84] García-Depraect O., Lebrero R., Martínez-Mendoza L.J., Rodriguez-Vega S., Aragão Börner R., Börner T., Muñoz R. (2023). Enhancement of Biogas Production Rate from Bioplastics by Alkaline Pretreatment. Waste Manag..

[85] Jin Y., Sun X., Song C., Cai F., Liu G., Chen C. (2023). Understanding the Mechanism of Enhanced Anaerobic Biodegradation of Biodegradable Plastics after Alkaline Pretreatment. Sci. Total Environ..

[86] Hobbs S.R., Parameswaran P., Astmann B., Devkota J.P., Landis A.E. (2019). Anaerobic Codigestion of Food Waste and Polylactic Acid: Effect of Pretreatment on Methane Yield and Solid Reduction. Adv. Mater. Sci. Eng..

[87] Modenbach A.A., Nokes S. (2014). Effects of Sodium Hydroxide Pretreatment on Structural Components of Biomass. Trans. ASABE.

[88] Ariunbaatar J., Panico A., Frunzo L., Esposito G., Lens P.N.L., Pirozzi F. (2014). Enhanced Anaerobic Digestion of Food Waste by Thermal and Ozonation Pretreatment Methods. J. Environ. Manag..

[89] Awasthi K., Akhtar S., Khan M.K.A. (2021). Bioplastic: An Accost towards Sustainable Development. NeuroPharmac J..

[90] Vardar S., Demirel B., Onay T.T. (2022). Degradability of Bioplastics in Anaerobic Digestion Systems and Their Effects on Biogas Production: A Review. Rev. Environ. Sci. Biotechnol..

[91] Venkiteshwaran K., Benn N., Seyedi S., Zitomer D. (2019). Methane Yield and Lag Correlate with Bacterial Community Shift Following Bioplastic Anaerobic Co-Digestion. Bioresour. Technol. Rep..

[92] Ashraf Joolaei A., Makian M., Prakash O., Im S., Kang S., Kim D.-H. (2024). Effects of Particle Size on the Pretreatment Efficiency and Subsequent Biogas Potential of Polylactic Acid. Bioresour. Technol..

[93] Nie R., Peng W., Lü F., Zhang H., Lu X., He P. (2024). Impact of the Thermo-Alkaline Pretreatment on the Anaerobic Digestion of Poly(butylene Adipate-Co-Terephthalate) (PBAT) and Poly(lactic Acid) (PLA) Blended Plastics. J. Hazard. Mater..

[94] Chang M., Li D., Wang W., Chen D., Zhang Y., Hu H., Ye X. (2017). Comparison of Sodium Hydroxide and Calcium Hydroxide Pretreatments on the Enzymatic Hydrolysis and Lignin Recovery of Sugarcane Bagasse. Bioresour. Technol..

[95] Jiang D., Ge X., Zhang Q., Zhou X., Chen Z., Keener H., Li Y. (2017). Comparison of Sodium Hydroxide and Calcium Hydroxide Pretreatments of Giant Reed for Enhanced Enzymatic Digestibility and Methane Production. Bioresour. Technol..

[96] Wang Y., Hu T., Zhang W., Lin J., Wang Z., Lyu S., Tong H. (2023). Biodegradation of Polylactic Acid by a Mesophilic Bacteria Bacillus Safensis. Chemosphere.

[97] Myburgh M.W., van Zyl W.H., Modesti M., Viljoen-Bloom M., Favaro L. (2023). Enzymatic Hydrolysis of Single-Use Bioplastic Items by Improved Recombinant Yeast Strains. Bioresour. Technol..

[98] Pooja N., Chakraborty I., Rahman M.H., Mazumder N. (2023). An Insight on Sources and Biodegradation of Bioplastics: A Review. 3 Biotech.

[99] Jiang X., Bi D., Cheng Y., Wang S., Peng B.-Y., Shen H., Zhang T., Xia X., Shen Z., Zhang Y. (2023). Enzyme Pretreatments for Anaerobic Co-Digestion of Food Waste Blended with Bioplastics: Effects on Methane Production and Microbial Community Structure. N. J. Chem..

[100] Liu W., Wang S., He S., Shi Y., Hou C., Jiang X., Song Y., Zhang T., Zhang Y., Shen Z. (2024). Enzyme Modified Biodegradable Plastic Preparation and Performance in Anaerobic Co-Digestion with Food Waste. Bioresour. Technol..

[101] Santos-Beneit F., Chen L.M., Bordel S., Frutos de la Flor R., García-Depraect O., Lebrero R., Rodriguez-Vega S., Muñoz R., Börner R.A., Börner T. (2023). Screening Enzymes That Can Depolymerize Commercial Biodegradable Polymers: Heterologous Expression of Fusarium Solani Cutinase in Escherichia Coli. Microorganisms.

[102] Preethi, Gunasekaran, Banu J.R. (2023). Indexing Energy and Cost of the Pretreatment for Economically Efficient Bioenergy Generation. Front. Energy Res..

[103] Divya D., Gopinath L.R., Merlin Christy P. (2015). A Review on Current Aspects and Diverse Prospects for Enhancing Biogas Production in Sustainable Means. Renew. Sustain. Energy Rev..

[104] Folino A., Pangallo D., Calabrò P.S. (2023). Assessing Bioplastics Biodegradability by Standard and Research Methods: Current Trends and Open Issues. J. Environ. Chem. Eng..

[105] Chinaglia S., Tosin M., Degli-Innocenti F. (2018). Biodegradation Rate of Biodegradable Plastics at Molecular Level. Polym. Degrad. Stab..

[106] Karki R., Chuenchart W., Surendra K.C., Shrestha S., Raskin L., Sung S., Hashimoto A., Kumar Khanal S. (2021). Anaerobic Co-Digestion: Current Status and Perspectives. Bioresour. Technol..

[107] Hoelzle R.D., Virdis B., Batstone D.J. (2014). Regulation Mechanisms in Mixed and Pure Culture Microbial Fermentation. Biotechnol. Bioeng..

[108] Kang J.-H., Kang S.-W., Kim W.-J., Kim D.-H., Im S.-W. (2022). Anaerobic Co-Digestion of Bioplastics and Food Waste under Mesophilic and Thermophilic Conditions: Synergistic Effect and Biodegradation. Fermentation.

[109] Maragkaki A., Tsompanidis C., Velonia K., Manios T. (2023). Pilot-Scale Anaerobic Co-Digestion of Food Waste and Polylactic Acid. Sustain. Sci. Pract. Policy.

[110] Cazaudehore G., Guyoneaud R., Lallement A., Souquet P., Gassie C., Sambusiti C., Grassl B., Jiménez-Lamana J., Cauzzi P., Monlau F. (2023). Simulation of Biowastes and Biodegradable Plastics Co-Digestion in Semi-Continuous Reactors: Performances and Agronomic Evaluation. Bioresour. Technol..

[111] Shafana Farveen M., Muñoz R., Narayanan R., García-Depraect O. (2025). Batch and Semi-Batch Anaerobic Digestion of poly(3-Hydroxybutyrate-Co-3-Hydroxyhexanoate) (PHBH) Bioplastic: New Kinetic, Structural, Microbiological and Digestate Phytotoxicity Insights. Sci. Total Environ..

[112] Zhou J., Ming S., Liu Q., Zhang Y., Duan N. (2024). Revealing the Synergy Mechanisms of Organic Components Anaerobic Co-Digestion from the Prevailing Tendency of Endogenous Inhibitors. Chem. Eng. J..

[113] Rajagopal R., Massé D.I., Singh G. (2013). A Critical Review on Inhibition of Anaerobic Digestion Process by Excess Ammonia. Bioresour. Technol..

[114] Gandhi B.P., Otite S.V., Fofie E.A., Lag-Brotons A.J., Ezemonye L.I., Semple K.T., Martin A.D. (2022). Kinetic Investigations into the Effect of Inoculum to Substrate Ratio on Batch Anaerobic Digestion of Simulated Food Waste. Renew. Energy.

[115] Puthumana A.B., Kaparaju P. (2024). Impact of Organic Load on Methane Yields and Kinetics during Anaerobic Digestion of Sugarcane Bagasse: Optimal Feed-to-Inoculum Ratio and Total Solids of Reactor Working Volume. Energies.

[116] Cioabla A.E., Ionel I., Dumitrel G.-A., Popescu F. (2012). Comparative Study on Factors Affecting Anaerobic Digestion of Agricultural Vegetal Residues. Biotechnol. Biofuels.

[117] García-Depraect O., Lebrero R., Rodriguez-Vega S., Börner R.A., Börner T., Muñoz R. (2022). Production of Volatile Fatty Acids (VFAs) from Five Commercial Bioplastics via Acidogenic Fermentation. Bioresour. Technol..

[118] Navaneethan N., Topczewski P., Royer S., Zitomer D. (2011). Blending Anaerobic Co-Digestates: Synergism and Economics. Water Sci. Technol..

